# Inhibition of Nuclear Transport of NF-ĸB p65 by the *Salmonella* Type III Secretion System Effector SpvD

**DOI:** 10.1371/journal.ppat.1005653

**Published:** 2016-05-27

**Authors:** Nathalie Rolhion, R. Christopher D. Furniss, Grzegorz Grabe, Aindrias Ryan, Mei Liu, Sophie A. Matthews, David W. Holden

**Affiliations:** Section of Microbiology, MRC Centre for Molecular Bacteriology and Infection, Imperial College London, London, United Kingdom; University of Toronto, CANADA

## Abstract

*Salmonella enterica* replicates in macrophages through the action of effector proteins translocated across the vacuolar membrane by a type III secretion system (T3SS). Here we show that the SPI-2 T3SS effector SpvD suppresses proinflammatory immune responses. SpvD prevented activation of an NF-ĸB-dependent promoter and caused nuclear accumulation of importin-α, which is required for nuclear import of p65. SpvD interacted specifically with the exportin Xpo2, which mediates nuclear-cytoplasmic recycling of importins. We propose that interaction between SpvD and Xpo2 disrupts the normal recycling of importin-α from the nucleus, leading to a defect in nuclear translocation of p65 and inhibition of activation of NF-ĸB regulated promoters. SpvD down-regulated pro-inflammatory responses and contributed to systemic growth of bacteria in mice. This work shows that a bacterial pathogen can manipulate host cell immune responses by interfering with the nuclear transport machinery.

## Author Summary

*Salmonella* Typhimurium replicates in macrophages through the action of effector proteins translocated into host cells by a type III secretion system (T3SS). We show that the T3SS effector SpvD targets the NF-ĸB pathway by interfering with nuclear translocation of p65. SpvD interacts with the exportin Xpo2. Perturbation of Xpo2 disrupts recycling of importin-α from the nucleus, leading to abrogation of p65 nuclear translocation. These data show that a bacterial pathogen manipulates host cell immune responses by interfering with nuclear transport machinery.

## Introduction

The NF-ĸB signalling pathway has a central role in the host response to infection by microbial pathogens, by stimulating innate and acquired host immune responses. Under normal physiological conditions, transcription factors of the NF-ĸB family such as p65 remain inactive in the cytoplasm through their interaction with inhibitors, the IĸBα proteins, which mask the nuclear localisation signal (NLS) of transcription factors. Following engagement of extracellular bacterial LPS by Toll-Like Receptor 4 (TLR4) or tumour necrosis factor (TNF) by the TNF receptor (TFNR), different pathways lead to phosphorylation and proteasomal degradation of IĸBα, allowing the NF-ĸB subunits to bind the adaptor protein importin-α (KPNA). This complex then interacts with one of up to 20 importin-β family members to enable nuclear transport through the nuclear pore complex. Within the nucleus, RanGTP binds to importin-β, dissociating the import complex and releasing the NF-ĸB subunits to initiate transcription of their target genes. Importin-β complexed with RanGTP is recycled to the cytoplasm while export of KPNA follows its interaction with the β-karyopherin exportin-2 (Xpo2, also called CAS) and RanGTP. Finally, cytoplasmic Ran GTPase activating protein (RanGAP) stimulates the Ran GTPase, generating RanGDP, which dissociates from the importins and thereby releases them for another import cycle [1,2].

Many pathogens, including *Salmonella enterica*, have acquired mechanisms that interfere with NF-ĸB signalling. Numerous components of the NF-ĸB signalling pathway are targeted by pathogen-mediated post-translational modifications (PTMs) that result in attenuation of the NF-ĸB dependent responses [3–9].

*Salmonella* has two type III secretion systems (T3SS) encoded within the *Salmonella* pathogenicity islands (SPIs) 1 and 2 that deliver virulence effector proteins into the host cell. The SPI-1 T3SS effectors are translocated across epithelial cell plasma membranes and mediate bacterial invasion and intestinal inflammation [10], while the SPI-2 T3SS translocates approximately 30 different effectors across the vacuolar membrane. Some of these maintain vacuolar membrane integrity and enable bacterial growth [11,12]. A few have been shown to interfere with host cell inflammatory responses. For example, SpvC has phosphothreonine lyase activity on MAPKs [13,14], SspH1 (translocated both by SPI-1 and SPI-2 T3SSs [15]) binds to the kinase PKN1 [16], which in turn regulates NF-ĸB and JNK signalling and AvrA (also translocated both by SPI-1 and SPI-2 T3SSs [17] inhibits NF-ĸB pathway in epithelial cells [18] via the JNK pathway [19] and tight junction stabilization [20]. PipA, GogA and GtgA redundantly target components of the NF-ĸB signaling pathway to inhibit transcriptional responses leading to inflammation [21].

Here, we used macrophages lacking TLR4 to reveal that the SPI-2 T3SS effector SpvD suppressed production of pro-inflammatory cytokines. We found that SpvD interfered with the NF-ĸB signalling pathway by preventing nuclear accumulation of p65. This was associated with nuclear accumulation of importin-α family members that are required for nuclear import of p65. Interestingly, SpvD interacted specifically with exportin Xpo2, which mediates nuclear-cytoplasmic recycling of importins. Together, this work reveals that a bacterial pathogen prevents host cell immune responses by interfering with nuclear transport machinery.

## Results

### Suppression of proinflammatory immune responses by SPI-2 T3SS effectors

**Fig 1:**
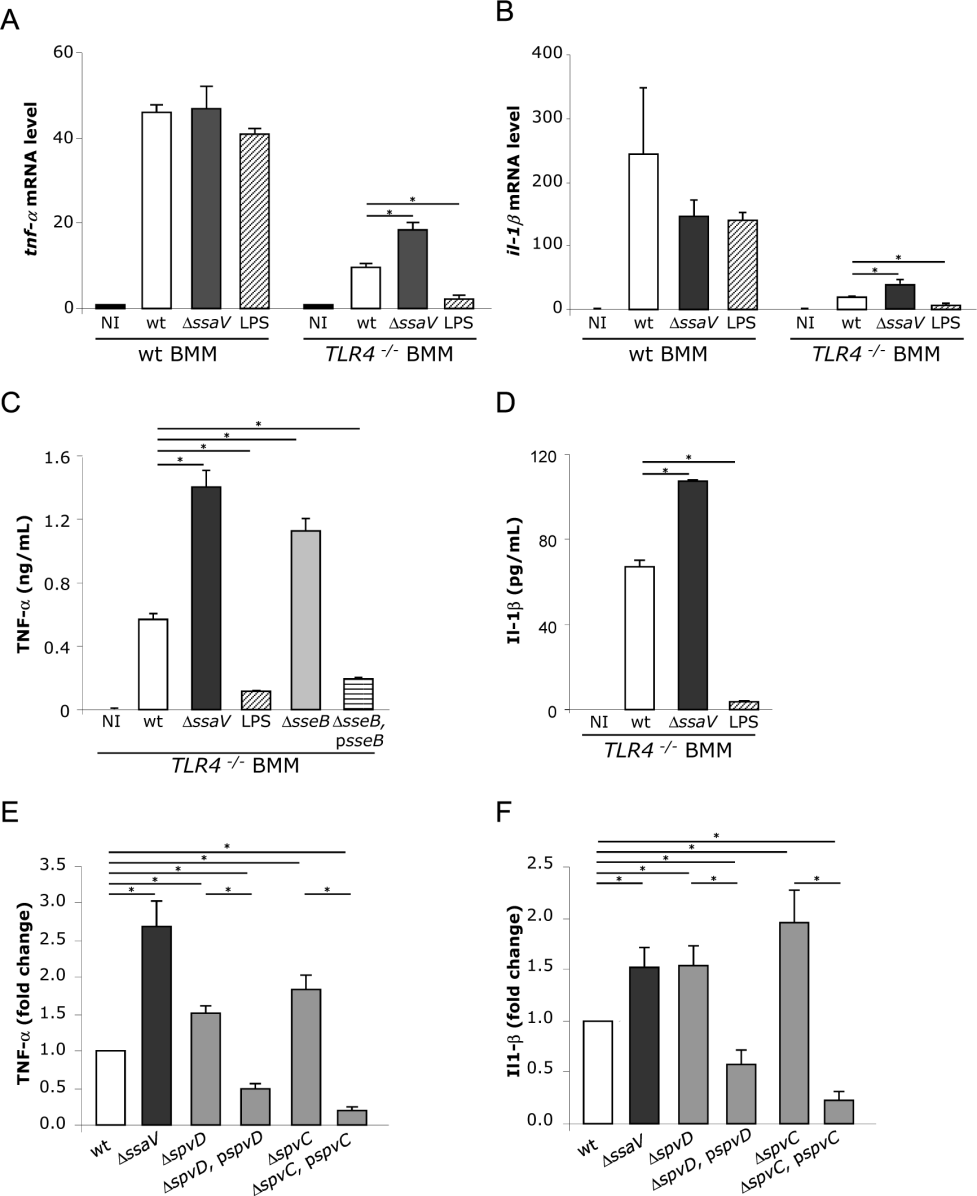
The SPI-2 T3SS reduces pro-inflammatory cytokine mRNA levels and protein secretion in *TLR4* ^-/-^ BMM. Wild-type and *TLR4* ^-/-^ BMMs were non-infected (NI), exposed to LPS (100 ng/ml) or infected with wild-type or Δ*ssaV* strains for 10 h and mRNA levels of *tnf*-*α* (A) and *il-1β* (B) were analysed. The transcript levels were normalized to the levels of *rsp9*, which were constant under all conditions used, and then expressed relative to those of NI wild-type BMMs. Results are expressed as mean ± SEM of at least 3 independent experiments. Levels of secreted TNF-α (C) and Il-1β (D) were quantified by ELISA in supernatants of non-infected (NI) *TLR4* ^-/-^ BMMs, *TLR4* ^-/-^ BMMs stimulated with LPS or *TLR4* ^-/-^ BMMs infected with indicated strains of *S*. Typhimurium at 10 h or 24 h post-uptake respectively. Results presented are the means ± SEM of triplicates of one experiment. These results are representative of three independent experiments and significant differences were observed in each experiment. P-values were obtained using two-tailed unpaired Student's t-test (*p < 0.05). Levels of secreted TNF-α at 10 h post-uptake (E) and Il-1β at 24 h post-uptake (F) were quantified by ELISA in supernatants of *TLR4* ^-/-^ BMMs infected with indicated strains of *S*. Typhimurium. The cytokine levels were expressed relative to those of BMMs infected with wild-type bacteria. Results are expressed as mean ± SEM of at least 3 independent experiments. Statistical significances were calculated using ANOVA followed by Bonferonni's multiple comparison test against wild-type strain.

We previously reported increases of more than 10-fold in mRNA for 26 genes in wild-type mouse bone marrow-derived macrophages (BMM) infected for 10 h by *S*. Typhimurium [22]. Many of these are likely to be due to the predominant effect of LPS on pro-inflammatory signalling pathways, since LPS and *Salmonella* cells induced similar changes in RAW 264.7 macrophages gene expression at 4 h post-challenge [23]. To analyse the effect of the SPI-2 T3SS in the context of TLR4-induced responses, we selected three genes (*tnf-α*, *il1-β* and *serpinB2*) that are induced by LPS [23,24]. Their mRNA levels were measured in BMM from wild-type and TLR4 knock-out (*TLR4* ^-/-^) mice, infected with wild-type or Δ*ssaV* (SPI-2 T3SS null mutant) *S*. Typhimurium for 10 h to allow sufficient time for SPI-2 T3SS effector translocation and activity, while minimising the difference in overall intracellular growth rate between wild-type and Δ*ssaV* mutant bacteria. The fold-increase in intracellular bacterial numbers (calculated from the ratio of the cfu at 10 h compared to 2 h post-uptake) was 2.20 ± 0.34 for the wild-type and 0.89 ± 0.09 for Δ*ssaV* mutant bacteria. Following infection of wild-type BMM for 10 h, the RNA levels of *tnf-α*, *il1-β* and *serpinB2* (quantified by real-time PCR (qRT-PCR)) increased by between 40 and 400 fold, and no significant differences were observed between BMM infected with wild-type or Δ*ssaV* mutant bacteria (**Fig 1A and 1B, S1 Fig**). Similar increases were detected following exposure of wild-type BMM to LPS, suggesting that TLR4 signalling accounted for the majority of these changes (**Fig 1A and 1B, S1 Fig**). However, *TLR4* ^-/-^ BMM infected with the Δ*ssaV* mutant had statistically significant higher levels of *tnf-α* and *il1-β* mRNA transcripts than BMM infected with wild-type bacteria (**Fig 1A and 1B**). No difference was observed for *serpinB2* mRNA transcripts levels (**S1 Fig**), indicating that transcription of not all the LPS-responsive genes was affected by the SPI2-T3SS. Levels of secreted TNF-α from *TLR4* ^-/-^ BMM infected with SPI-2 null mutants (Δ*ssaV* or Δ*sseB*) were also significantly greater than from *TLR4* ^-/-^ BMM infected with wild-type bacteria or a complemented SPI-2 null mutant (Δ*sseB*, p*sseB*) (**Fig 1C**). The level of secreted Il1-β was below the threshold of detection at 10 h post-bacterial uptake but was also reduced in a SPI-2 T3SS-dependent manner at 24 h post-bacterial uptake (**Fig 1D**). Together these results provide evidence that the SPI-2 T3SS suppresses pro-inflammatory cytokine mRNA levels and protein secretion in *TLR4* ^-/-^ macrophages. The failure to detect such differences using wild-type BMM presumably reflects the potent effect of LPS on pro-inflammatory signalling pathways in this experimental system.

### SpvD is a SPI-2 T3SS effector suppressing cytokine secretion

To identify SPI-2 T3SS effector(s) involved in down-regulation of pro-inflammatory immune responses, several SPI-2 T3SS mutant strains (representing single mutants lacking individual effectors) [25] were screened for levels of secreted TNF-α from *TLR4* ^-/-^ BMM at 10 h post-uptake (**S1 Table**). Of these, 2 strains (Δ*spvC* and Δ*spvD*) induced significantly more cytokine secretion from infected BMM compared to BMM infected by the wild-type strain (**Fig 1E and 1F**). SpvC and SpvD are encoded by the *spvRD* operon (composed of *spvA*, *B*, *C* and *D*, and regulated by *spvR* [26] on the *Salmonella* virulence plasmid (pSLT)) and SpvC is a phosphothreonine lyase that interferes with MAPK signalling [14]. BMM infected with deletion mutants carrying the low copy number plasmid pACYC184 containing the corresponding wild-type allele of the gene (Δ*spvC*, p*spvC* or Δ*spvD*, p*spvD*) produced significantly less TNF-α and Il1-β than BMM infected with the single mutant strains (**Fig 1E and 1F**). To analyse if the effects of SpvD are confined to macrophages, Hela cells were infected with *Salmonella* strains for 8, 12 or 24 h and levels of IL-8 in supernatants were quantified by ELISA. Infection with wild-type bacteria induced IL-8 production but no increased production was detected in cells infected with the Δ*spvD* mutant or the Δ*spvC* mutant (as shown in [14]) (**S2 Fig**). However, cells infected with the Δ*spvD*, p*spvD* or Δ*spvC*, p*spvC* strains showed significantly less IL-8 production compared with the wild-type strain at all time-points tested (P ≤ 0.05 for all time points). Therefore, the effects of SpvD occur both in macrophages and another cell type that does not normally respond to LPS.

SPI-2 T3SS-dependent secretion of SpvD into minimal medium and its translocation into host cells (using a CyaA fusion protein) were shown previously [29]; however its function is unknown and its contribution to virulence of *S*. Typhimurium in mice is not clear [27–30]. To confirm SPI-2 T3SS-dependent secretion and translocation of SpvD, strains producing double-HA tagged SpvD from pSLT were used for an *in vitro* secretion assay and for macrophage infection. Secretion of SpvD-2HA from bacteria grown in minimal medium was investigated following shift of ambient pH from 5.0 to 7.2, which stimulates effector secretion from the SPI-2 T3SS [31]. A similar amount of SpvD-2HA was present in lysates from wild-type and Δ*ssaV* mutant bacteria (**S3A Fig**). SpvD-2HA was also detected in the secreted fraction from wild-type bacteria but not from the Δ*ssaV* mutant. To investigate SpvD translocation into host cells, RAW macrophages were infected for 22 h with *Salmonella* strains producing SpvD-2HA. Cells were then fixed and labelled to detect *Salmonella* and the HA tag. Translocated SpvD-2HA was not detected within infected macrophages by immunofluorescence microscopy under these conditions. However when cells were incubated for 2 h prior to fixation with the proteasome inhibitor MG132, weak but distinct and reproducible cytoplasmic labelling of SpvD-2HA was detected in the cytoplasm of the majority of cells infected with wild-type but not Δ*ssaV* mutant *Salmonella* (**S3B Fig**). These results suggest that relatively small amounts of SpvD are translocated into the host cell cytoplasm.

**Fig 2:**
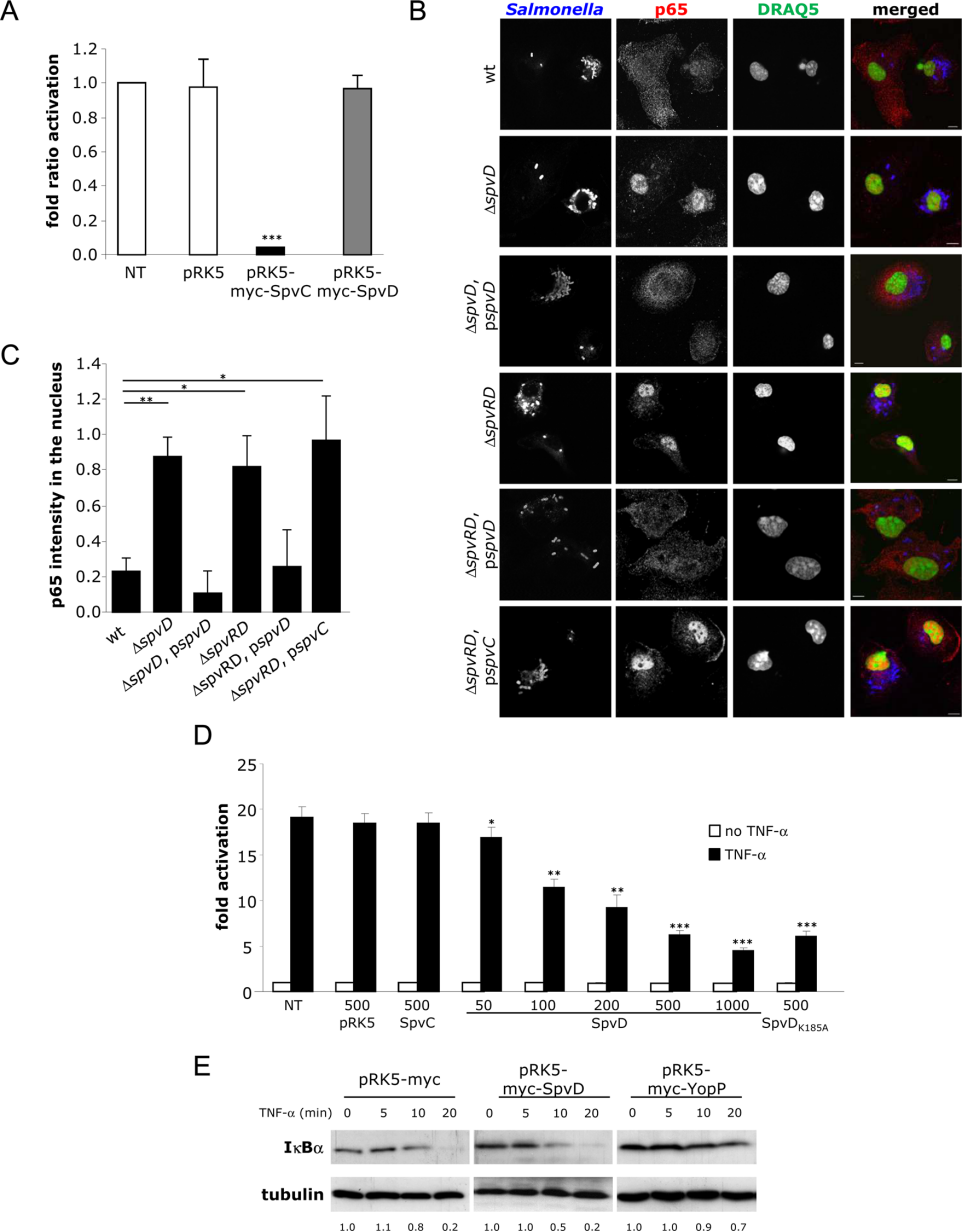
SpvD prevents nuclear accumulation of p65 but not degradation of IĸBα. (A) SpvD does not inhibit activation of an AP-1-regulated promoter. HEK-293 cells were cotransfected with an AP-1-dependent luciferase reporter plasmid, pTK-Renilla luciferase and pRK5myc, pRK5myc-SpvC or pRK5myc-SpvD. Cells were then stimulated with PMA for 6 h. Firefly luciferase activity was normalized against Renilla luciferase activity. Results are expressed as fold induction compared to non-transfected (NT) cells. Values are expressed as mean ± SEM of 3 independent experiments and P-values were obtained using two-tailed unpaired Student's t-test (*** p < 0.005). (B-C) SpvD prevents nuclear translocation of p65. (B) Representative immunofluorescence fields of p65 localisation using anti-p65 (red) in *TLR4* ^-/-^ BMMs infected for 10 h with indicated *Salmonella* strains (blue). Cell nuclei were stained with DRAQ5 (green). Scale bar, 8 μm. (C) Quantification above background levels of p65 intensity in the nucleus was analyzed by three-dimensional (3D) confocal microscopy. Background levels were determined using signals measured in uninfected cells. P-values were obtained using two-tailed unpaired Student's t-test (*p < 0.05; ** p < 0.01). (D) SpvD inhibits activation of an NF-ĸB -regulated promoter. HEK-293 cells were co-transfected with an NF-ĸB -dependent luciferase reporter plasmid, pTK-Renilla luciferase and pRK5myc, pRK5myc-SpvC, pRK5myc-SpvD or pRK5myc-SpvD_K185A_ at the indicated amounts (in ng). The NF-ĸB pathway was activated with TNF-α and luciferase activity was measured after 8 h. Results are expressed as fold activation in relation to unstimulated and non-transfected (NT) cells. Values are expressed as mean ± SEM of at least 3 independent experiments and statistical significances were calculated using ANOVA followed by Bonferonni's multiple comparison test against NT cells (*p < 0.05; ** p < 0.01; *** p < 0.005). (E) Degradation of IĸBα is not affected by SpvD. HEK-293 cells transfected by pRK5myc-SpvD or pRK5myc-YopP were prepared at the indicated times after TNF-α stimulation and analysed by SDS-PAGE and immunoblotting with anti- IĸBα and anti-tubulin antibodies. Ratio of IĸBα normalised to unstimulated cells is indicated below immunoblots.

Since SpvC inhibits MAP kinase signalling [14] we tested if SpvD affects this pathway in cells expressing a luciferase reporter gene under the control of MAPK-dependent AP-1 binding sequences. HEK 293 cells were co-transfected with vectors expressing myc-SpvD or myc-SpvC (as positive control) or myc vector alone (empty pRK5 vector), along with vector expressing the luciferase reporter under the control of AP-1 promoter and vector constitutively expressing the Renilla luciferase, as an internal control for normalisation of data. Cells were exposed to PMA for 6 h to stimulate MAP kinase pathways. Then cells were lysed and bioluminescence was measured to determine the fold-difference of MAP kinase activation in relation to non-transfected cells. Immunoblots of cell lysates following SDS-PAGE, using anti-myc and anti-tubulin antibodies, confirmed that similar numbers of cells expressing similar levels of effectors were analysed. Ectopic expression of SpvC resulted in a strong inhibition of luciferase activity, while transfection of empty vector or of vector producing SpvD did not inhibit luciferase activity (**Fig 2A**), suggesting that unlike SpvC, SpvD does not inhibit the MAP kinase signalling pathway.

### SpvD prevents nuclear accumulation of p65

Some T3SS effectors inhibit pro-inflammatory cytokine production and secretion by interfering with proteins of the NF-ĸB pathway [6]. To determine the potential effect of SpvD on nuclear translocation of the NF-ĸB transcription factor p65, confocal microscopy was used to quantify the amounts of nuclear p65 in *TLR4* ^-/-^ BMM infected with different *Salmonella* strains at 10 h post-bacterial uptake. Cells were immunolabelled with anti-*Salmonella* and anti-p65 antibodies and nuclei were stained with the DNA dye, DRAQ5 (**Fig 2B**). Three-dimensional image projections were acquired randomly and nuclear p65 intensity was quantified using Volocity software. The intensity of p65 in the nuclei of infected *TLR4* ^-/-^ BMM was significantly increased when SpvD was absent (Δ*spvD*, Δ*spvRD* (corresponding to Δ*spvR*-*spvA*-*spvB*-*spvC*-*spvD* mutant strain) or Δ*spvRD*,p*spvC* strains) and was restored to wild-type levels by mutants carrying p*spvD* (Δ*spvD*,p*spvD* or Δ*spvRD*,p*spvD* strains) (**Fig 2C**), indicating that of *spv*-encoded proteins, only SpvD prevents nuclear accumulation of p65. NleC, a T3SS effector of enteropathogenic *E*. *coli* is a protease that cleaves p65 directly [32–35]. Therefore, we analysed total cell levels of p65 in *TLR4* ^-/-^ BMM infected with different *Salmonella* strains at 10 h post-uptake. No significant differences were detected (**S4A Fig**). To determine if the influence of SpvD on p65 localisation was cell-type specific, its effects on p65 and its localisation were analysed during infection of HeLa epithelial cells. The amount of p65 in total cell lysates and in nuclei of HeLa cells infected with *Salmonella* strains was quantified by immunoblot after cell fractionation. No differences were detected in total cell extracts, but the amount of p65 in the nuclei of cells infected with the Δ*spvD* mutant was consistently increased compared to wild-type infected cells (**S4B Fig**). The amount of nuclear p65 was restored to wild-type levels in cells infected with the Δ*spvD* mutant carrying a plasmid containing *spvD* (**S4B Fig**). In addition, total levels of p65 in HeLa cells transfected with pRK5myc-SpvD, pRK5myc-SpvC (used as a negative control) or pRK5myc-NleC (used as a positive control) were analysed by FACS after labelling of the cells with anti-p65 and anti-myc antibodies. Levels of p65 were determined in myc-positive cells and normalised to non-transfected cells. Degradation of p65 occurred only in cells expressing NleC (**S4C and S4D Fig**). These results indicate that SpvD reduces nuclear translocation of p65 in both mouse macrophages and human epithelial cells, but does not affect the overall cellular pool of p65. The amounts of two other immune related transcription factors (p50 and STAT2) in total cell lysates and in nuclei of HeLa cells infected with *Salmonella* strains were quantified by immunoblot after cell fractionation. No differences were detected in total cell extracts, but the amount of p50 in the nuclei of cells infected with the Δ*spvD* mutant was consistently increased compared to wild-type -infected cells (**S4B Fig**). The amount of nuclear p50 was restored to wild-type levels in cells infected with the Δ*spvD* mutant carrying a plasmid containing *spvD*. No differences in STAT2 levels in nuclei or total cell lysates were detected. These results indicate that SpvD specifically targets the import of NF-ĸB -associated proteins.

The predicted product of *spvD* is a hydrophilic protein of 216 amino acids [36] and visual alignment of a region of SpvD with several prokaryotic phosphothreonine lyases [37] revealed a region in SpvD (between amino acids 181 to 213) that is conserved among these enzymes (**S4E Fig**). This area contains an invariant Lys residue (K185) and mutation of this residue in SpvC results in a loss of phosphothreonine lyase activity [38]. As a further test of the effect of SpvD on the NF-ĸB signalling pathway, and to investigate the importance of K185, HEK 293 cells were co-transfected with a reporter plasmid encoding luciferase under the control of NF-ĸB promoter and a plasmid encoding either myc alone (empty pRK5 vector), myc-SpvC (as a negative control), myc-SpvD or myc-SpvD_K185A_ (SpvD carrying a lysine-to-alanine substitution at residue 185). Luciferase activity was assayed 8 h after TNF-α stimulation and normalised to non-transfected and unstimulated cells. Non-transfected cells underwent an approximately 20-fold increase in activation following stimulation with TNF-α when compared to resting cells. Transfection of 500 ng of empty vector or vector expressing SpvC did not prevent NF-ĸB activation, whereas expression of SpvD or SpvD_K185A_ reduced activation by approximately 65% (**Fig 2D**). Inhibition of NF-ĸB activation by SpvD was observed after transfection of 50 ng of DNA and increased with increasing amounts of DNA. Together, these results indicate that SpvD is sufficient to block the activation of an NF-ĸB -regulated promoter and that K185 is not required for SpvD activity.

To investigate if SpvD interferes with IĸBα degradation induced upon stimulation with TNF-α, HEK 293 cells were transfected with plasmids encoding either myc alone (empty vector), myc-SpvD or myc-YopP (a T3SS effector of *Yersinia enterolitica* that inhibits IĸBα degradation [39–41], used here as a positive control), then stimulated with TNF-α for 5, 10 or 20 min to induce IĸBα degradation. Proteins in cell extracts were then analyzed by SDS-PAGE and immunoblotting. As expected, degradation of IĸBα was inhibited in cells expressing myc-YopP (**Fig 2E**). However, IĸBα was degraded in cells transfected with the empty vector or with the plasmid encoding myc-SpvD (**Fig 2E**). Because only approximately 60% of the cell population was efficiently transfected (as measured by microscopic examination), a potential effect of SpvD on IĸBα degradation might be difficult to detect when analysing the whole cell population by immunoblotting. Therefore, IĸBα degradation following TNF-α stimulation was also analysed by flow cytometry after labelling of the cells with anti- IĸBα and anti-myc antibodies. Levels of IĸBα were quantified in myc-positive cells and normalised to non-stimulated cells. TNF-α -induced IĸBα degradation was only prevented in cells expressing YopP (**S4F Fig**), indicating that SpvD does not inhibit IĸBα degradation and that it must prevent nuclear accumulation of p65 by interfering with the NF-ĸB pathway after IĸBα degradation.

### Effect of SpvD on importin-α

**Fig 3:**
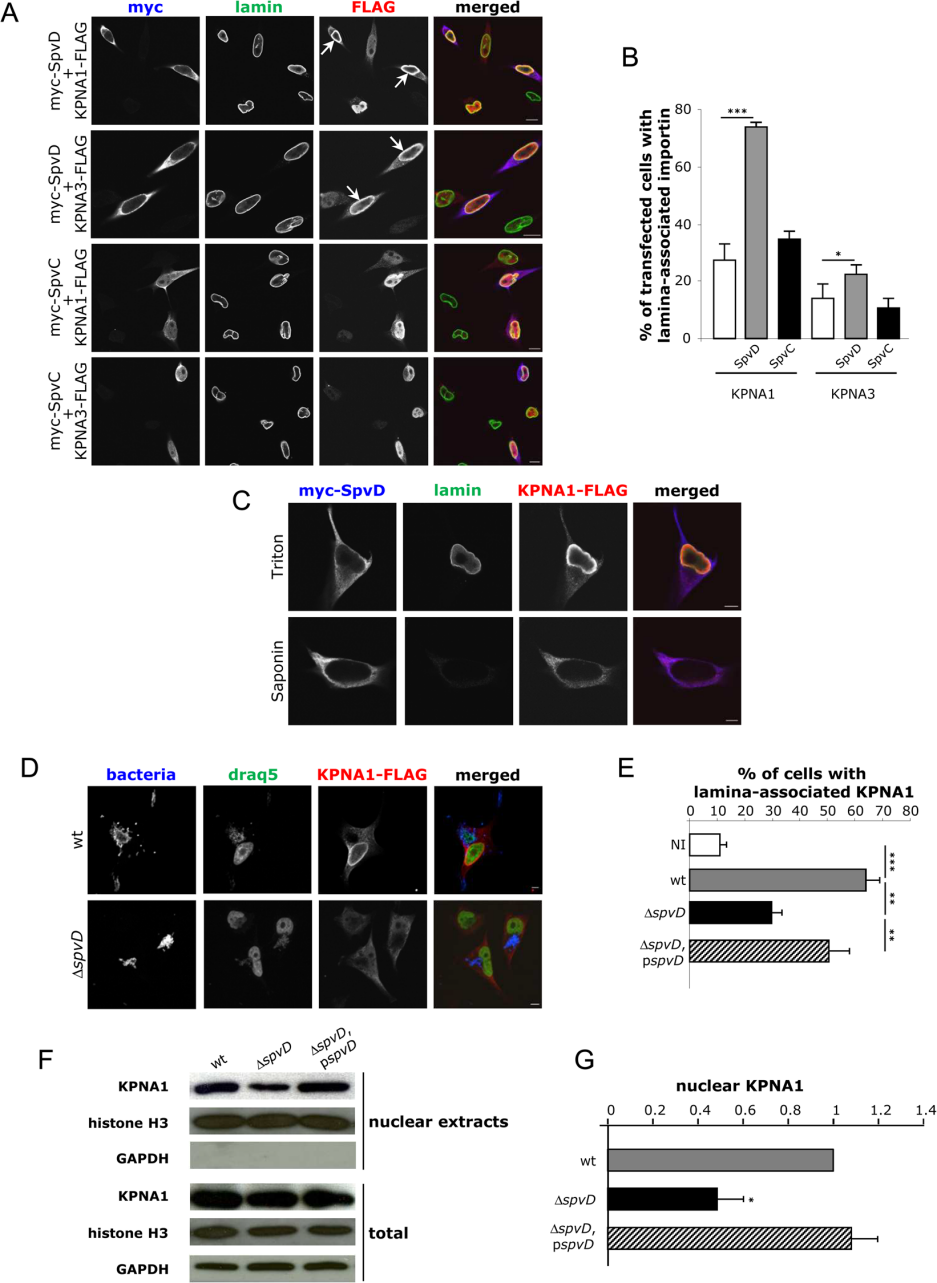
SpvD induces an intra-nuclear accumulation of importins. (A) HeLa cells were cotransfected with FLAG-KPNA1 or FLAG-KPNA3 and pRK5myc-SpvD or pRK5myc-SpvC. Samples were fixed, permeabilised in Triton X-100, labelled with anti-FLAG (red), anti-myc (blue) and anti-lamin (green) antibodies and analysed by confocal microscopy. Scale bar, 10 μm. White arrows indicate nuclear lamina-associated KPNAs. (B) Quantification of cells with nuclear lamina-associated KPNA1 or KPNA3 after transfection with pRK5myc-SpvD or pRK5myc-SpvC. Values are expressed as mean ± SEM of at least 4 independent experiments (*p < 0.05; *** p < 0.005). (C) HeLa cells were cotransfected with FLAG-KPNA1 and pRK5myc-SpvD. Samples were fixed, permeabilised in Triton X-100 or Saponin and labelled as in A. Scale bar, 8 um. (D) HeLa cells were cotransfected with FLAG-KPNA1 plasmids then infected for 14 h with *Salmonella* strains. Samples were fixed, permeabilised in Triton X-100 and labelled with anti-FLAG (red) and anti-*Salmonella* (blue) antibodies. Cell nuclei were stained with DRAQ5 (green). Scale bar, 10 μm. (E) Quantification of cells with nuclear lamina-associated KPNA1 after infection with *Salmonella* strains. Values are expressed as mean ± SEM of at least 4 independent experiments (** p < 0.01; *** p < 0.005). (F) HeLa cells were infected for 14 h with *Salmonella* strains. Nuclear and total cell extracts were analysed by SDS-PAGE and immunoblotting with anti-KPNA1, anti-histone H3 and anti-GAPDH antibodies. (G) Ratio of KPNA1 in the nucleus normalised to wild-type -infected cells. Values are expressed as mean ± SEM of 3 independent experiments. P-values were obtained using two-tailed unpaired Student's t-test (*p < 0.05).

Translocation of p65 into the nucleus requires proteins of the karyopherin (KPNA) family [42,43], also called importin-α. To determine the potential effect of SpvD on KPNA1 and KPNA3, two importin-α proteins known to interact with p65 [43–45], confocal microscopy was used to analyse their localisation in HeLa cells. Since attempts to detect endogenous KPNA1 or KPNA3 using commercial antibodies failed, HeLa cells were co-transfected with plasmids encoding either KPNA1-FLAG or KPNA3-FLAG and plasmids encoding myc-SpvD or myc-SpvC (as a control). Cells were immunolabelled with anti-FLAG, anti-myc and anti-lamin antibodies. Consistent with the localisation of SpvD following its translocation from bacteria (**S3B Fig**), SpvD was found in the host cell cytoplasm after transfection (**Fig 3A**). In addition to nuclei containing diffuse labelling of KPNA1-FLAG or KPNA3-FLAG, in some nuclei, the labelling was predominantly in the region of the nuclear envelope, where it colocalised partially with lamin (**Fig 3A**). This lamina-associated labelling was observed in all transfected cell populations, but its frequency was enhanced 2.7-fold by KPNA1 (P<0.001) and 1.6-fold by KPNA3 (P<0.05) when SpvD was co-expressed (**Fig 3B**). In addition, the enhanced frequency of lamina-associated labelling was not observed when SpvC was co-expressed (**Fig 3B**), indicating that it is specific to SpvD. To determine if the reorganisation of KPNA1 induced by myc-SpvD was localised inside or outside the nucleus, differential membrane permeabilisation was done using either Triton X-100 (under conditions in which it permeabilises both the plasma and nuclear membranes) or Saponin (at a concentration where it permeabilises only the plasma membrane). SpvD-induced accumulation of KPNA1 at the nuclear envelope was only observed when cells were permeabilised with Triton X-100, indicating that this importin accumulates within the nucleus (**Fig 3C**).

To analyse the effect of SpvD on importin-α localisation during infection, HeLa cells transfected with plasmids encoding KPNA1-FLAG or KPNA3-FLAG were infected for 14 h with *Salmonella* strains. Cells were immunolabelled with anti-*Salmonella* and anti-FLAG antibodies and nuclei stained with DRAQ5. Deletion of SpvD led to a significant decrease of the percentage of cells with nuclear lamina-associated KPNA1 (**Fig 3D and 3E**) or KPNA3 (**S5 Fig**). Cells infected with the deletion mutant carrying a plasmid-borne wild-type allele of *spvD* induced a greater proportion of these structures than the deletion mutant (**Fig 3E**; p<0.01). These structures might be an artefact of overexpression of KPNA1 and KPNA3 but do suggest an SpvD-dependent accumulation of KPNA1 and KPNA3 in the nucleus. To analyse the effect of SpvD on the localisation of endogenous KPNA1, the amount of endogenous KPNA1 in nuclei of HeLa cells infected with *Salmonella* strains was quantified by immunoblotting after cell fractionation. A significant decrease in the amount of endogenous KPNA1 in the nuclei of cells infected with the Δ*spvD* mutant was detected, compared to wild-type *Salmonella*-infected cells (**Fig 3F and 3G**). The amount of nuclear KPNA1 was restored to that of the wild-type level in cells infected with the Δ*spvD* mutant carrying a plasmid containing *spvD* (**Fig 3F and 3G**). Therefore, in addition to inhibiting nuclear accumulation of p65, SpvD also causes nuclear accumulation of KPNA1.

### SpvD interacts with Xpo2

Recycling of KPNAs from the nucleus to the cytoplasm is mediated by Xpo2 (also named CAS or CSE1) [46]. To confirm that depletion of Xpo2 inhibits recycling of KPNAs, confocal microscopy was used to analyse the localisation of KPNA1-FLAG in HeLa cells depleted of Xpo2 (by siRNA of Xpo2, **S6A Fig**) and then transfected with a plasmid encoding KPNA1-FLAG. Cells were immunolabelled with anti-Xpo2 and anti-FLAG antibodies and the percentage of cells having detectable cytoplasmic KPNA1 were scored. siRNA of Xpo2 in HeLa cells caused accumulation of KPNA1 in the nucleus (**S6B Fig**) and a decrease in the percentage of cells having detectable KPNA1 in the cytoplasm (**S6C Fig**), compared to non-treated cells or cells exposed to a scrambled siRNA. However, the nuclear KPNA1 that was observed in the absence of Xpo2 was not localised at the lamina, as was observed in the presence of SpvD.

**Fig 4:**
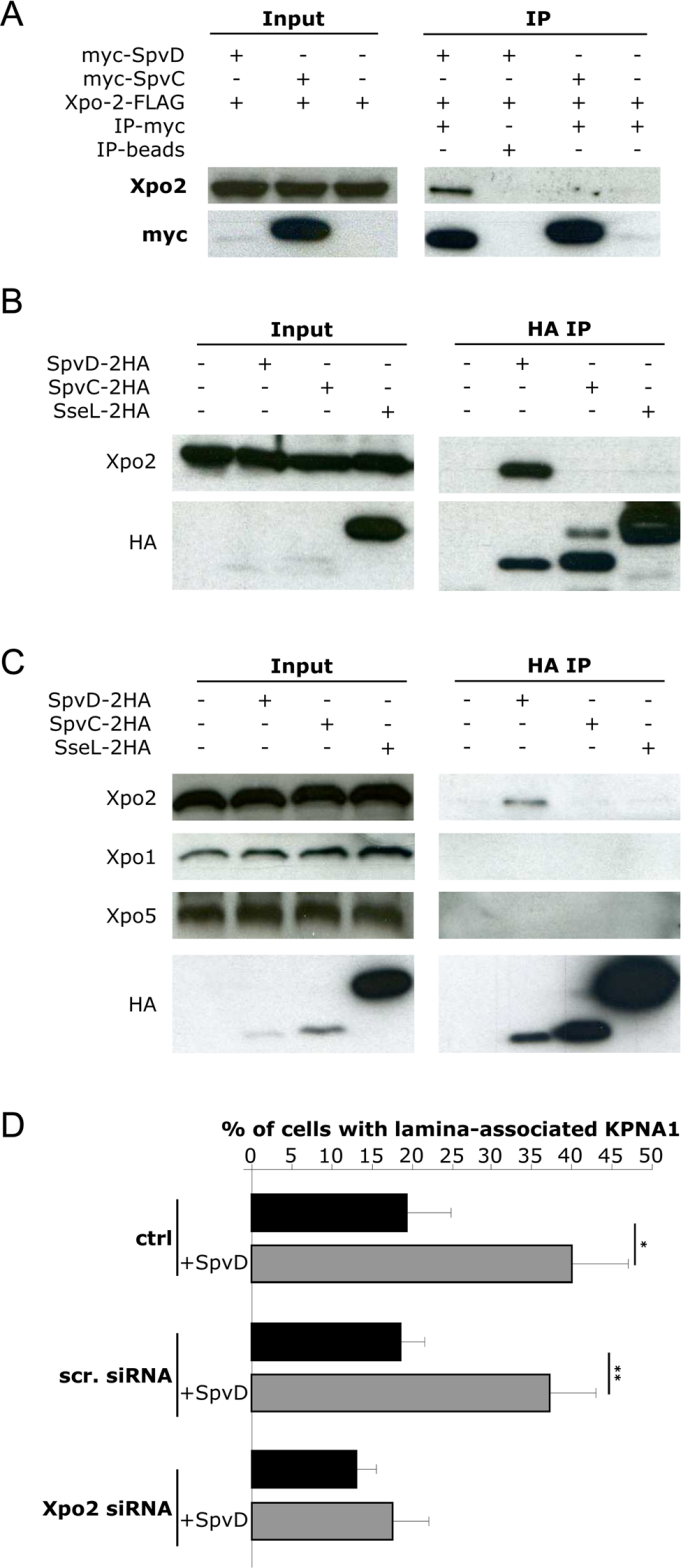
**SpvD interacts with Xpo2** (A) HEK cells transfected with vectors expressing Xpo2-FLAG and myc-SpvD or myc-SpvC were lysed and proteins were immunoprecipitated with anti-myc antibody-conjugated beads, or beads as a control. Xpo2-FLAG, myc-SpvD and myc-SpvC were detected in input samples (input) and after immunoprecipitation (IP) by means of SDS-PAGE and immunoblotting. (B) RAW macrophages or (C) HeLa cells were infected for 20 h with wild-type *Salmonella* expressing either SpvD-2HA, SpvC-2HA or SseL-2HA. Proteasome inhibitor MG132 was added to the culture medium for the last 2 h of infection. Cells were then lysed and proteins were immunoprecipitated with HA antibody. SDS-PAGE blots were probed for HA and endogenous Xpo2, Xpo1 or Xpo5. (D) HeLa cells depleted of Xpo2 (Xpo2 siRNA) or treated with scramble siRNA (scr. siRNA) and then transfected with FLAG-KPNA1 and myc-SpvD were fixed and labelled with anti-myc and anti-FLAG antibodies. Intra-nuclear rings of KPNA1 were quantified blindly by microscopy. Values are expressed as mean ± SEM of 3 independent experiments and P-values were obtained using two-tailed unpaired Student's t-test (*p < 0.05; ** p < 0.01).

Knock-down of Xpo2 in HeLa cells was reported to cause a decrease of p65 translocation after stimulation with TNF-α [47]. To determine the effect of inhibition of Xpo2 expression on p65 localisation in our experiments, confocal microscopy was used to analyse localisation of p65 in HeLa cells depleted of Xpo-2 and stimulated with TNF-α for 45 min. Cells were immunolabelled with anti-Xpo-2 and anti-p65 antibodies and the intensity of p65 within the nucleus was quantified using Volocity software. siRNA of Xpo2 in HeLa cells led to a decrease in levels of nuclear p65 after stimulation with TNF-α compared to control cells (non-treated or scrambled siRNA) (**S6D and S6E Fig**). Since cytoplasmic SpvD and inhibition of Xpo2 expression both led to nuclear accumulation of KPNA1 and reduced levels of nuclear p65, we hypothesized that SpvD might interact with Xpo2. To investigate this, HEK cells were co-transfected with a plasmid encoding Xpo2-FLAG and a plasmid encoding either myc-SpvD or myc-SpvC. Cells were lysed and proteins immunoprecipitated with anti-myc antibody-conjugated beads. Immunoblot analysis revealed that SpvC failed to interact with Xpo2 but that SpvD and Xpo2 formed a complex (**Fig 4A**). To determine if SpvD interacts with endogenous Xpo2 in infected cells, RAW macrophages and HeLa cells were infected with *Salmonella* expressing either SpvD-HA or two other effectors translocated by the SPI-2 T3SS (SpvC-HA or SseL-HA), followed by immunoprecipitation using anti-HA conjugated beads. Input and output samples were analysed by SDS-PAGE and immunoblotting with anti-Xpo2 and anti-HA antibodies. SpvD bound to endogenous Xpo2 following its translocation from intracellular bacteria (**Fig 4B and 4C**). No binding was observed when cells were infected with *Salmonella* expressing SpvC-2HA or SseL-2HA despite the fact that these two effectors were evidently more abundant than SpvD-2HA (**Fig 4B and 4C**). Xpo1 and Xpo5 are additional members of the exportin family, have some structural similarities to Xpo2 and mediate the export of proteins with a nuclear export signal and of RNA respectively [48]. However, when membranes were probed with anti-Xpo1 and anti-Xpo5 antibodies, no interaction between SpvD-2HA and these proteins was detected (**Fig 4C**). To analyse if the interaction of Xpo2 with SpvD affects the cellular localisation of Xpo2, the amount of Xpo2 in total cell lysates and in cytoplasm of HeLa cells infected with *Salmonella* strains was quantified by immunoblotting after cell fractionation. No cytoplasmic increase of Xpo2 in wild-type -infected cells was detected (**S7A Fig**). In addition, HeLa cells were transfected with plasmids encoding myc-SpvD or myc-SpvC (as control) and the amount of Xpo2 in total cells lysates and in cytoplasm was quantified by immunoblotting after cell fractionation (**S7B Fig**). No cytoplasmic increase of Xpo2 in cells transfected with plasmid encoding myc-SpvD was detected, suggesting that SpvD does not sequester Xpo2 in the cytoplasm.

These results show that SpvD localises to the host cell cytoplasm, inhibits nuclear accumulation of p65, causes nuclear accumulation of KPNA1 and KPNA3, and binds specifically to Xpo2. To determine if nuclear lamina-associated KPNA1 induced by SpvD is dependent on Xpo2, confocal microscopy was used to analyse the localisation of KPNA1-FLAG in HeLa cells depleted of Xpo2 and then transfected with a plasmid encoding KPNA1-FLAG with or without a second plasmid encoding myc-SpvD. Cells were immunolabelled with anti-FLAG and anti-myc antibodies and the number of cells with nuclear lamina-associated KPNA1-FLAG were counted (**Fig 4D**). When SpvD was present, the frequency of lamina-associated KPNA1 was significantly enhanced by 2.1-fold in non-treated HeLa cells and by 2.0-fold in cells exposed to a scrambled siRNA. However, no statistical difference was observed in HeLa cells depleted of Xpo2. Together, these results indicate that the ability of SpvD to induce lamina-associated KPNA1 is dependent on the presence of Xpo2.

### Effect of SpvD on virulence and cytokine expression in mice

**Fig 5:**
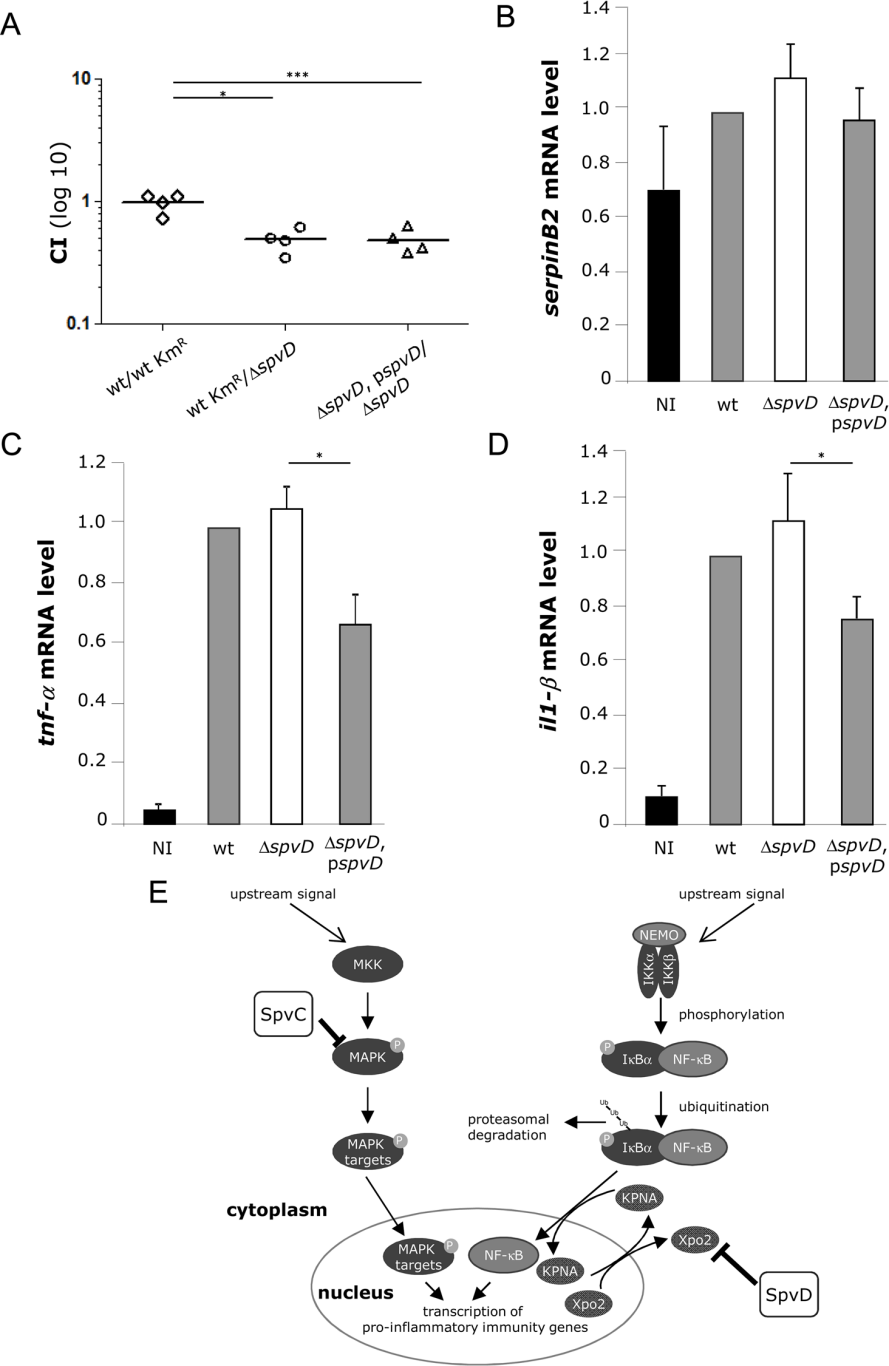
Effect of SpvD on virulence and cytokine expression *in vivo*. (A) Lack of SpvD causes virulence attenuation of *Salmonella* in the mouse model of systemic infection. C57BL/6 mice were inoculated by intraperitoneal injection (i.p.) with equal numbers (2.5 x 10^4^ CFU) of indicated bacteria. Bacteria were recovered from infected spleens 3 days post-inoculation. CI values were calculated as described in materials and methods. The scatter plot displays values obtained for individual mice and the mean is indicated (line) (*p < 0.05; *** p < 0.005). (B-D) C57BL/6 mice were inoculated by i.p. with indicated bacteria, RNAs from splenic macrophages were recovered at 2 days post-inoculation and *serpinB2* (B), *tnf*-α (C) and *il1-*β (D) mRNA levels were analysed by qRT-PCR. The transcript levels were normalized to the levels of *rsp9*, which were constant under all conditions used, and then expressed relative to those of wild-type-infected mice. Results are expressed as mean ± SEM of at least 3 independent experiments. P-values were obtained using two-tailed unpaired Student's t-test (*p < 0.05). (E) Model depicting pathways targeted by SpvC and SpvD to overcome PAMP-induced defences in host cells. Immunoblots are representative of three independent experiments.

Previous work failed to reveal a clear and consistent role of SpvD in virulence *in vivo* [27–30]. To reassess the possibility of an effect of SpvD on systemic growth of bacteria, mice were inoculated by intraperitoneal injection of a mixture of equivalent cfu of kanamycin resistant wild-type bacteria and the Δ*spvD* mutant strain. After 72 h of infection, bacteria were quantified after plating spleen homogenates on LB medium and LB medium containing kanamycin to distinguish the strains. The competitive index (CI) for the Δ*spvD* mutant was 0.48 ± 0.11 (P≤0.05) when compared to wild-type (**Fig 5A**). To verify that this attenuation was due to loss of SpvD, a second experiment was conducted in which mice were infected as above but with a mixture of Δ*spvD* mutant and the same strain carrying *spvD* allele on the pACYC plasmid. The CI for this combination was 0.47 ± 0.11 (P≤0.005) (**Fig 5A**), showing that the *spvD* allele rescues the growth defect of the mutant, thereby confirming that SpvD contributes to systemic virulence in the mice.

To assess the effect of SpvD on LPS-induced genes in mice, RNA levels of *serpinB2*, *tnf-α* and *il1-β* in infected splenic macrophages of C57BL/6 mice inoculated with *Salmonella* strains were compared by qRT-PCR (**Fig 5B**–**5D**). For *serpinB2* mRNA transcripts, no significant differences were detected between mice infected with different *Salmonella* strains (**Fig 5B**). No significant differences in *tnf-α* and *il1-β* were detected between mice infected with the wild-type or Δ*spvD* mutant bacteria, but macrophages infected with Δ*spvD*, p*spvD* bacteria contained fewer *tnf-α* and *il1-β* transcripts **(Fig 5C and 5D),** indicating that SpvD can depress cytokine expression *in vivo*.

## Discussion

As a basis for identifying SPI-2 T3SS effectors that interfere with host proinflammatory immune signalling, we analysed the effect of the SPI-2 T3SS on mRNA levels of *tnf-α* and *il1-β* in wild-type BMM at 10 h post-bacterial uptake. No obvious differences were detected, suggesting that either SPI-2 effectors do not have a significant effect on macrophage mRNA levels or that their activities are masked by the massive changes induced by LPS [23] and possibly other PAMPs. Use of *TLR4* ^-/-^ BMMs provided evidence that the SPI-2 T3SS does suppress pro-inflammatory cytokine production. Among several SPI-2 T3SS effector mutants that were tested subsequently, infection with either Δ*spvC* or Δ*spvD* strains led to increased *tnf-α* and *il1-β* secretion. SpvD and SpvC-dependent effects also occurred in HeLa cells, showing that their functions are not confined to macrophages. The effect of the Δs*pvC* mutant was unsurprising, since two previous studies have indicated an anti-inflammatory effect of SpvC, caused by its phosphothreonine lyase activity on ERK1/2, p38 and JNK [14,49].

SpvD was identified in a proteomic study as being secreted by both the SPI-1 and SPI-2 T3SSs and its translocation into J774 macrophage-like cells was confirmed using a CyaA fusion protein assay [29]. Using an epitope-tagged version under the control of its own promoter in the virulence plasmid, we showed that SpvD is secreted into the culture medium in a SPI-2 T3SS-dependent manner. SPI-2 T3SS-dependent translocation of SpvD into the cytoplasm of infected cells was also confirmed by immunofluorescence microscopy, but only when cells had been treated with a proteasome inhibitor. In addition, bacterially translocated SpvD-2HA was barely detectable in the input sample from infected HeLa cells and RAW macrophages, whereas SpvC-2HA and SseL-2HA were clearly more abundant (**Fig 4B and 4C**). This indicates that SpvD is translocated in relatively small amounts and/or is more prone to degradation compared to other effectors. Despite some amino acid similarity in a region containing the catalytic lysine of SpvC, SpvD did not require the corresponding lysine to suppress TNF-dependent activation of an NF-ĸB reporter. Furthermore, following transfection, SpvC but not SpvD inhibited activation of an AP-1-dependent promoter, showing that these effectors are likely to have distinct biochemical activities and physiological effects.

Several other bacterial effectors have been shown to interfere with NF-ĸB signalling. In most cases they affect the degradation of IĸBα and/or the release of p65 [4–6]. For example, the *Shigella flexneri* kinase OspG interacts with host ubiquitin-conjugating enzymes to abrogate IĸBα degradation and thus blocks nuclear translocation of p65 [50]. NleC of enteropathogenic and enterohaemorrahgic *E*. *coli* is a metalloprotease that degrades both free cytosolic p65 and p65 when complexed with IĸBα [33–35]. Abrogation of p65 translocation has also been described for the effectors NleB and NleE from enteropathogenic *E*. *coli* and OspZ from *Shigella flexneri* [51,52] NleE is an unusual methyltransferase that modifies crucial cysteines in the zinc finger domains in ubiquitin-chain sensory proteins TAB2 and TAB3 and thereby disrupts NF-ĸB signalling [53]. NleB is a N-acetylglucosamine transferase whose action blocks signalling from the TNFR and its associated adapters [54–56]. We found that SpvD prevents nuclear accumulation of p65 by interfering with the NF-ĸB pathway after IĸBα degradation but it did not affect overall cellular levels of p65. SpvD might not be the only effector that inhibits the nuclear translocation of p65 following IĸBα degradation. Indeed, the *Bordetella* effector BopN [57] localises to the nucleus and appears blocks nuclear translocation of p65 without affecting IĸBα degradation; however, its host target(s) remain to be identified. SpvD might specifically target the import of NF-ĸB -associated proteins as it also prevents nuclear accumulation of NF-ĸB subunit p50 but not of STAT2.

There are 7 different importin-α (KPNA) isoforms and several have the capacity to interact with p65. Fagerlund et al. reported an interaction between p65 and both KPNA4 (α3) and KPNA3 (α4) but not KPNA2 (α1) [43,44], while Cunningham et al. showed that p65 binds to KPNA2 [42], Liang et al. reported that nuclear translocation of p65 relies mainly on KPNA2 [47] and Sun et al. showed that p65 translocation in leucocytes does not involve KPNA4, but rather KPNA1 (α5) [45]. p65 might use different isoforms depending on host cell type, time and strength of pathway activation and other conditions. Furthermore, *in vitro* binding assays with purified proteins or lysates might not reflect normal physiological interactions. However, our results showing that SpvD affects nuclear accumulation of both p65 and p50 are consistent with its effect on KPNA4 (α3), which mediates nuclear import of both transcription factors [43].

We were unable to distinguish endogenous KPNAs by confocal microscopy using commercial antibodies and therefore cells were transfected with plasmids encoding FLAG-tagged KPNAs and anti-FLAG antibodies were used to detect them by confocal microscopy. Following transfection or bacterial translocation, SpvD stimulated a nuclear lamina-associated accumulation of overexpressed KPNA1 and KPNA3. No effect was observed on KPNA2 or KPNA4 localisation. We are unaware of other bacterial proteins that cause a similar effect, but Hantaan virus nucleocapsid protein N inhibits p65 nuclear translocation by binding directly to KPNAs [58]. We were unable to obtain evidence for an interaction between SpvD and KPNA1, 2, 3 or 4 by co-immunoprecipitation assays. Instead, following transfection or bacterial translocation, SpvD interacted specifically with endogenous Xpo2, which is required for recycling of KPNAs from the nucleus to the cytoplasm [46]. Inhibition of Xpo2 expression led to nuclear accumulation of KPNA1 and also reduced nuclear translocation of p65 ([47] and this work). Despite apparently stable binding between SpvD and Xpo2, Xpo2 localisation was not noticeably affected by SpvD. It is possible that SpvD is an enzyme that modifies Xpo2. However, following immunoprecipitation by SpvD-2HA after infection of macrophages, mass spectrometric analysis of Xpo2 did not reveal any post-translational modifications such as phosphorylation, acetylation or conjugation by ubiquitin and ubiquitin-like proteins. It is possible that such modifications did occur but are relatively transient, labile or did not survive the extraction or ionization processes. Alternatively SpvD might affect Xpo2 function non-enzymatically, or use Xpo2 as a docking site or cofactor to mediate an effect on another component of the importin/exportin machinery. A link between binding of SpvD to Xpo2 and the effect of SpvD on KPNA1 was established by showing that the SpvD-induced accumulation of KPNA1 at the nuclear lamina was dependent on Xpo2. Sequestration of KPNA in the nuclear envelope has been observed in cells treated with histone deacetyltransferase inhibitors [59]. However, this treatment also resulted in accumulation of Xpo2 in nuclear aggregates [59], which are clearly distinct from the effects of SpvD.

Due to the lack of clear and consistent data on the contribution of SpvD to *Salmonella* virulence [27–30], we reassessed its effect on systemic growth of bacteria in mice. Our evidence indicates that SpvD does contribute to systemic virulence in the mouse. The disparity might be attributable to the different mouse strains used in each study. We did not detect a significant difference in the levels of *tnf-α* and *il1-β* mRNA in splenic macrophages of wild-type mice infected with the wild-type or *spvD* mutant strains. However, the complemented mutant in which SpvD was produced from a plasmid caused a significant reduction of *tnf-α* and *il1-β* mRNA levels in splenic macrophages of wild-type mice. It seems likely that the effects of SpvD produced by wild-type bacteria are subtle and can be masked by other immune-modulating bacterial molecules present in these relatively short-term infection assays, as evidenced by the initial experiments using wild-type macrophages.

Together, our evidence suggests that an interaction between SpvD and Xpo2 disrupts the nuclear/cytoplasm cycling of KPNA1 and KPNA3, which interferes with nuclear translocation of p65 and activation of NF-ĸB regulated promoters (**Fig 5E**). Whatever the precise mechanism, it is remarkable that *Salmonella* has evolved the means to inhibit the two main pro-inflammatory signalling pathways that are activated following detection of bacterial pathogens through two proteins (SpvC and SpvD) that are encoded by adjacent genes on the same operon (**Fig 5E**).

## Materials and Methods

### Bacterial strains and plasmids

The *S*. Typhimurium strains (wild-type 12023 and its mutant derivatives) and plasmids used in this study are listed in **S2 Table**. Primers used for construction of all strains or plasmids are listed in **S3 Table**. Bacteria were grown in Luria Bertani (LB) medium at 37°C with shaking and supplemented with ampicillin (50 μg/ml), kanamycin (50 μg/ml) or chloramphenicol (34 μg/ml) as appropriate. *S*. Typhimurium mutant strains were constructed using a one-step λ Red recombinase chromosomal inactivation system [60]. Plasmid pKD4 was used as the template to amplify the kanamycin (km) resistance gene and amplification reaction products were transferred into pKD46-containing bacteria expressing λ Red recombinase by electroporation. To excise the km resistance marker, the mutant strains were transformed with pCP20 helper plasmid expressing the FLP recombinase. Virulence plasmid-encoded *spvD* was double hemagglutinin (HA)-tagged using the procedure described previously [61]. To obtain pACYC*spvD*-2HA, the double-HA tagged *spvD* was amplified from the virulence plasmid by PCR and cloned into pACYC184. To obtain pRK5-myc-SpvD, pRK5-myc-SpvC, pRK5-myc-YopP and pRK5-myc-NleC, the genes of interest were amplified by PCR and cloned into pRK5-myc. To obtain pRK5-myc-SpvD_K185A_, site-directed mutagenesis was performed by inverse PCR using pRK5-myc-SpvD as DNA template. Lysine at residue 185 of SpvD was changed to Alanine using SpvD_K185A_1 and SpvD_K185A_2. To create pcDNA3.1*Xpo2*-FLAG, the pCDNA3.1*Xpo5*-FLAG construct [62] was digested by *NheI* and *XhoI* restriction enzymes to obtain pcDNA3.1 vector backbone. In parallel, total cellular RNA was extracted from HeLa cells using the Qiagen RNeasy mini kit following manufacturer’s instructions. RNA were reverse transcribed to cDNA using the SuperScript II Reverse Transcriptase kit (Invitrogen) and Xpo2, encoded by the gene *CSE1L*, was amplified with the Expand Long Template PCR system (Roche) from total cDNA using primers CAS *NheI* and CAS *XhoI* and cloned into pcDNA3.1. Obtained plasmids were verified by sequencing.

### Antibodies

The following antibodies were used for immunofluorescence, FACS and immunoblot analysis: goat anti-*Salmonella* (CSA-1, Kirkegaard and Perry Laboratories), rabbit anti-DnaK [63], rat anti-HA (3F10, Roche), mouse anti-HA (HA11, Covance), mouse anti-FLAG (M2, Sigma-aldrich), rabbit anti-myc (Cell Signaling), mouse anti-β tubulin (Sigma-aldrich), rabbit polyclonal anti-GAPDH (Abcam), rabbit or goat anti-p65 (Santa Cruz), mouse anti- IĸBα (Cell Signaling), rabbit polyclonal anti-lamin B1 (Abcam), rabbit polyclonal anti-Xpo2 (CSE1L, Abcam), rabbit anti-Xpo1 (CRM1, Santa Cruz), rabbit anti-Xpo5 (Sigma-aldrich), rabbit anti-histone H3 (Abcam), rabbit anti-KPNA1 (Proteintech), mouse anti-p50 (Biolegend) and rabbit anti-STAT2 (Santa Cruz). Alexa Fluor 488-, 555- and 633- conjugated donkey anti-rat, anti-mouse, anti-goat and anti-rabbit antibodies were from Life technologies, UK.

### pH shift and protein secretion assay

Protein secretion assays were done as described previously [31]. Briefly, bacterial strains were grown overnight in LB broth and sub-cultured in MgM-MES medium pH 5.0 for 4 h to assemble and activate SPI-2 T3SS. Bacterial cells were then collected by centrifugation, re-suspended into MgM-MES at pH 7.2 and incubated at 37°C for 1.5 h.

### Cell culture

HeLa (human epithelial cell line) cells, HEK (human embryonic kidney) cells and RAW264.7 macrophages used in this study were obtained from the European Collection of Animal and Cell Cultures (Salisbury, UK) and maintained in Dulbecco’s modified Eagle’s medium (DMEM) (Life technologies) supplemented with 10% foetal calf serum (FCS) (PAA Laboratories or Sigma-Aldrich) at 37°C in 5% CO_2_. Primary bone-marrow macrophages (BMM) were obtained from C57BL/6 wild-type (Charles River) or *TLR4* ^-/-^ mice (kind gift from Prof Maria Belvisi and Dr Mark Birrell (Respiratory Pharmacology, National Heart and Lung Institute, Imperial College, London)). BMM were grown in RPMI (Life Technologies) supplemented with 10% FCS, 2 mM glutamine, 1 mM sodium pyruvate, 10 mM HEPES, 50 μM β-mercaptoethanol, 100 U/ml penicillin/streptomycin (Sigma-Aldrich), and L929 cell-conditioned medium 20% (vol/vol; National Institute for Medical Research). After 3 days of culture, further fresh complete medium containing L929-cell conditioned medium was added to the growing macrophages. On day 7, cells were washed and seeded in complete medium without antibiotic and incubated for 24 h before bacterial challenge.

### DNA transfection

HeLa cells were seeded in 24-well plates at a concentration of 2 x 10^4^ cells/well 24 h before transfection with Lipofectamine 2000 (Life technologies) following the manufacturer’s protocol. Unless indicated, 100 ng of DNA were used. Cells were used 24 h after transfection.

### siRNA transfection

The siRNA oligo duplex targeted against Xpo2 was custom synthesized using a previously described targeting sequence [64] (ThermoScientific). The scramble control oligos (ThermoScientific) were designed not to target any human mRNA transcript.

HeLa cells were seeded in 24-well plates at a concentration of 5 x 10^4^ cells/well and grown until they were 70% confluent. siRNA transfection using RNAiMAX (Life technologies) was carried out according to the manufacturer’s protocol with a final concentration of siRNA oligos of 10 nM. A scramble oligo sequence was included in all experiments as a negative control. Cells were used 72 h after siRNA transfection.

### Bacterial infection of cells

HeLa cells and RAW264.7 macrophages were infected with *S*. Typhimurium strains as described previously [65]. BMM were infected as described previously [66].

### TNF-α stimulation

HeLa cells were seeded into 24-well plates at a concentration of 2 x 10^4^ cells/well 24 h before stimulation. Unless indicated, cells were stimulated with TNF-α (10 ng/mL) for 45 min at 37°C. For IĸBα degradation analysis, at indicated times after TNF-α stimulation, cells were washed and either lysed for immunoblotting analysis or trypsinised for flow cytometry assays.

### Fixation, permeabilisation, fluorescence labelling and microscopy

All samples were fixed in 3% paraformaldehyde (PFA) and unless indicated, permeabilised in 0.2% Triton-X100 for 6 min. All antibodies were diluted to the appropriate concentrations in PBS containing 10% horse serum. The coverslips were washed twice in PBS, incubated with primary antibodies for 1 h, washed 3 times in PBS, incubated with secondary antibodies for 30 min and stained with the nucleic acid dye, DRAQ5 (Alexis) for 20 min. For specific plasma membrane permeabilisation, all antibodies were diluted in PBS containing 10% horse serum and 0.1% saponin (Fisher) and after fixation with PFA, cells were washed twice in PBS, incubated with primary antibodies for 1 h, washed 3 times in PBS and incubated with secondary antibodies for 30 min. Coverslips were washed and mounted onto glass slides using Mowiol mounting medium. Cells were analysed using either an epi-fluorescence microscope (BX50; Olympus) or a confocal laser-scanning microscope (LSM510 or LSM710; Zeiss GmBH). All scorings were done blindly and at least 100 cells were analysed per coverslip. To determine p65 intensity in the nucleus, confocal three-dimensional Z-stacks were acquired for each sample using a 63x objective with a slice of increment of 0.5 μm. At least 50 infected cells were imaged for each condition. Rendered three-dimensional stacks were analysed with Volocity image analysis software (Perkin Elmer).

### Cell lysis, cell fractionation and immunoblotting

HeLa cells were collected from a well in a 24-well plate 3 days after transfection with siRNAs. Infected BMM were collected from a well in a 24-well plate at 10 h post-bacterial uptake. For IĸBα degradation analysis, at indicated times after TNF-α stimulation, cells were washed and collected after trypsinisation. Cells were lysed in 5x sample buffer (0.25 M Tris-Cl pH 6.8, 10% SDS, 10% β-Mercaptoethanol, 10% glycerol, 0.05% Bromophenol Blue). Samples were boiled for 5 min and separated by SDS-PAGE. Samples were then transferred onto PVDF Immobilon-P membranes (Millipore), and Immunoblotting was carried out according to the manufacturer’s instructions. Immunoblots shown in each figure are representative of three independent experiments.

For cell fractionation, 5 x 10^6^ cells were collected, washed twice with cold PBS and lysed in cold hypotonic buffer (10 mM HEPES pH 7.9, 10 mM KCl, 0.1 mM EDTA, 0.1 mM EGTA, 1 mM DTT) supplemented with complete protease inhibitor cocktail without EDTA (Roche) on ice for 15 min. NP-40 was added to a final concentration of 0.625% and cells were vortexed vigorously for 10 s. Samples were centrifuged for 30 s at 16000 *g* and the supernatants were harvested as cytoplasmic fraction. The nuclear pellets were then resuspended in cold hypertonic buffer (20 mM HEPES pH 7.9, 0.4 M NaCl, 1 mM EDTA, 1 mM EGTA, 1 mM DTT) supplemented with complete protease inhibitor cocktail (Roche). The samples were incubated at 4°C for 15 min with agitation. The supernatants were collected after centrifugation for 5 min at 16000 *g* as nuclear proteins. Cytoplasmic and nuclear proteins were frozen in 5x sample buffer at -20°C until use.

### Flow cytometry quantification of cellular protein levels

For flow cytometry assays to determine protein levels by immunolabelling, trypsinised cells were washed twice in PBS prior to fixation in 3% PFA for 20 min at room temperature. Cells were then washed twice in PBS and permeabilised in 0.08% Triton X-100 for 10 min. Cells were incubated for 1 h at room temperature with primary antibodies diluted in PBS. Cells were then washed twice and incubated for 30 min with secondary antibodies diluted in PBS. Finally, cells were washed and resuspended in PBS. Flow cytometry was performed on a two-laser, four colour FACSCalibur cytometer (BD Biosciences) using Cell Quest Pro software. For each sample, 45,000–50,000 events were analysed. Collected data were analysed with FlowJo 8.1.1 software (Treestar).

### RNA extraction, microarray analysis and qRT-PCR

Cells subjected to LPS (1 μg/ml) stimulation for 2 h were used as controls. At 10 h post-uptake, BMM were washed and RNA was isolated using TRIzol according to the manufacturer’s directions (Invitrogen). Contaminating genomic DNA was removed using DNaseI (Qiagen). RNAs (400 ng) were reverse transcribed with Quantiscript Reverse transcriptase (QuantiTect Reverse Transcription kit, Qiagen) for 25 min at 42°C. Quantification of the mRNA levels was done using SensiMix dT kit (Quantace) and specific primers (**S3 Table**) on Rotor-Gene 3000 (Corbett Research).

### Enzyme-linked immunosorbent assay

For TNF-α and IL1-β quantification, at 10 h or 24 h post-bacterial uptake or after 2 h of LPS (1 μg/ml) stimulation, supernatants from infected BMM were collected, centrifuged and stored at −80°C. For IL-8 quantification, at 8 h, 12 h or 24 h post-bacterial uptake, supernatants from infected HeLa cells were collected, centrifuged and stored at −80°C. The amount of TNF-α, IL1-β or IL-8 released in the culture supernatant was determined by enzyme-linked immunosorbent assay (ELISA; R&D Systems) and cytokine concentrations were assessed according to the manufacturer's instructions.

### Luciferase reporter assays

HEK293 cells were seeded at a density of 5 x 10^4^ cells per well in a 24-well plate 16 h prior to transfection. Cells were transfected for 16 h with 50 ng of luciferase reporter plasmid (AP-1 or NF-ĸB dependent luciferase reporter plasmid), 30 ng of pTK-Renilla luciferase, and 100 ng of expression vectors (myc-effector or myc vector alone). For MAPK reporter assays, cells were then incubated with 25 ng/ml of PMA for 6 h and harvested in 100 μl of passive lysis buffer (Promega). For NF-ĸB reporter assays, cells were then incubated with 10 ng/ml of TNF-α for 8 h and harvested in 100 μl of passive lysis buffer (Promega). Luciferase activity was measured using Dual Luciferase reporter assay system (Promega) and a TD20/20 Luminometer (Turner Designs) and normalised according to Renilla luciferase intensity. The data presented are from at least three independent experiments.

### Immunoprecipitation

HEK cells were seeded at a density of 2 x 10^5^ cells per well in a 6-well plate 16 h prior to transfection. Cells were transfected with 1 μg of indicated DNA with Lipofectamine 2000. Three wells were used per condition. Cells were lysed 24 h post-transfection in GTPase lysis buffer (50 mM Tris Cl pH 7.4, 150 mM NaCl, 5 mM MgCl_2_, 0.5% Triton X-100) supplemented with complete protease inhibitor cocktail without EDTA (Roche) on ice for 30 min. Lysates were then centrifuged at 16000 *g* and the supernatants were harvested and incubated with 25 μl of anti-myc conjugated beads for 2 h at 4°C. Samples were then centrifuged at 1500 *g* and supernatant was removed. Pellets were washed 3 times with 50 mM Tris Cl pH 7.4. Samples were then separated by SDS-PAGE and analysed by immunoblot.

HeLa cells and RAW macrophages were grown on 150 mm dishes (Corning) seeded at a concentration of 4x10^6^ cells and 8x10^6^ per dish, respectively. Two dishes were used per condition. Cells were then infected with *S*. Typhimurium strains expressing either SpvD-2HA, SpvC-2HA or SseL-2HA for 18 h. Proteasome inhibitor MG132 (10 μg/ml) was added to the culture medium for 2 h prior to cell lysis. Cells were lysed in GTPase lysis buffer supplemented with complete protease inhibitor cocktail without EDTA (Roche) on ice for 30 min. Lysates were then centrifuged at 16000 *g* and the supernatants were harvested and incubated with 25 μl of anti-HA beads (Pierce) for 2 h at 4°C. Samples were then placed on a magnetic rack and supernatant was removed. Pellets were washed 3 times with 5 mM Tris Cl pH 7.4, 15 mM NaCl, 0.5 mM MgCl_2_, 0.05% Triton X-100. Proteins bound to anti-HA beads were eluted using HA-peptide (Sigma-Aldrich) and were then separated by SDS-PAGE and analysed by immunoblotting.

### Mice infections

To prepare the inocula, bacteria were first grown overnight in LB broth and then subcultured at a dilution of 1:100 for a further 2 h. Cultures were diluted to a concentration of 2.5 × 10^4^ cfu/ml in physiological saline.

For competitive index (CI) measurements, bacterial cultures were mixed for intra-peritoneal inoculation (0.2 mL per mouse). Viable bacteria in inocula were quantified by dilution and plating onto LB agar plates with appropriate antibiotics to distinguish between strains. Female C57BL/6 mice (Charles River, 6–12 weeks) were sacrificed at 3 days post inoculation. The spleens were removed aseptically and homogenized in distilled water by mechanical disruption. Serial dilutions were plated on LB agar for cfu enumeration. Strains were distinguished by differential counting or replica plating on antibiotic-supplemented plates. For each mouse, the CI was calculated by dividing the output ratio (i.e. strain a *versus* strain b) divided by the input ratio. The log CI values were used to calculate means and for statistical analyses. A wild-type strain carrying a kanamycin resistance cassette on the chromosome (STM0857, [14]) was used to represent wild-type bacteria in the CI assay. Its CI was 0.97 ± 0.18 (**Fig 5A**) when compared to wild-type bacteria, indicating that kanamycin resistance cassette does not affect virulence.

For mRNA extraction, mice were infected by intra-peritoneal inoculation with 1 × 10^4^ bacteria and sacrificed 48 h post-inoculation. Spleens were harvested, homogenized and splenic CD11b(+) cells enriched using magnetic beads following manufacturer’s instructions (Milteneyi Biotec). RNA of purified cells was isolated using TRIzol according to the manufacturer’s directions (Invitrogen). mRNA reverse transcription and qRT-PCR were done as described above.

### Ethics statement

Animals were used in accordance with UK Home Office regulations. The Imperial College Animal Welfare and Ethical Review Body (AWERB) committee approved the project licence for animal research (70/7768). The following people formed the panel: Applicant Scientist, CBS site manager / NACWO, NVS, Peer Scientist and a Lay person.

### Statistical analysis

All results are reported as mean ± Standard Error of the Mean (SEM). Statistical analyses were done using two-tailed unpaired Student’s t-test or ANOVA followed by Bonferonni's multiple comparison test. Differences denoted in the text as significant fall below a p-value of 0.05.

## Supporting Information

S1 Fig The SPI-2 T3SS does not affect mRNA levels of all the LPS-responsive genes. Wild-type and *TLR4* ^-/-^ BMMs were non-infected (NI), exposed to LPS (100 ng/ml) or infected with wild-type or Δ*ssaV* strains for 10 h and mRNA levels of *serpinB2* were analysed by qRT-PCR after reverse transcription of RNA extracted from cells. The transcript levels were normalized to the levels of *rsp9*, which were constant under all conditions used, and then expressed relative to those of non-infected wild-type BMMs. Results are expressed as mean ± SEM of at least 3 independent experiments.
(TIF)

S2 Fig SpvD and SpvC reduce pro-inflammatory cytokine secretion in HeLa cells. Levels of secreted IL-8 at 8 h, 12 h or 24 h post-uptake were quantified by ELISA in supernatants of HeLa cells, infected with indicated strains of *S*. Typhimurium. The cytokine levels were expressed relative to those of HeLa cells infected with wild-type bacteria. Results are expressed as means ± SEM of 3 independent experiments and P-values were obtained using two-tailed unpaired Student's t-test. (*p < 0.05; ** p < 0.01; *** p <0.005).
(TIF)

S3 Fig SpvD-2HA is secreted and translocated into infected macrophages in SPI-2 T3SS-dependent manner. (A) Secretion of SpvD-2HA upon pH shift. Strains producing double-HA tagged SpvD from the virulence plasmid were used for pH shift analysis (as described in materials and methods). Secreted fractions and whole cell lysates were subjected to SDS-PAGE and immunoblotting. The intrabacterial protein DnaK was used as control. (B) RAW macrophages were infected for 24 h with *Salmonella* strains producing SpvD-2HA from the *pSLT* virulence plasmid; 10 μg/ml of MG132 was added to the culture media for the last 2 h of infection. Samples were fixed and labelled with antibodies against *Salmonella* and SpvD-2HA (green and red, respectively, on merged images). Scale bar, 2 μm.
(TIF)

S4 Fig SpvD does not affect cellular p65 levels and acts downstream of IĸBα. (A) *TLR4* ^-/-^ BMMs were infected with different strains of *Salmonella* as indicated and p65 levels assessed by immunoblotting at 10 h post uptake. The same membrane was probed for GADPH as a loading control. Ratio of p65 normalised to wild-type-infected cells is indicated below immunoblots. (B) HeLa cells were infected for 14 h with *Salmonella* strains. Nuclear and total cell extracts were analysed by SDS-PAGE and immunoblotting with anti-histone H3, anti-GAPDH, anti-p65, anti-p50 and anti-STAT2 antibodies. Ratio of p65, p50 and STAT2 normalised to wild-type-infected cells are indicated below immunoblots. (C) Representative flow cytometry histogram of levels of p65 in HeLa cells non transfected (red) or transfected by pRK5myc-SpvD (blue), pRK5myc-SpvC (brown) or pRK5myc-NleC (green). (D) Quantification of p65 in cells expressing SpvD, SpvC or NleC. Data were normalised to non-transfected control cells (NT). Results are expressed as means ± SEM of 3 independent experiments and P-values were obtained using two-tailed unpaired Student's t-test. (** p < 0.01, compared to NT). (E) Alignment of SpvD C-terminal sequence (amino acids 181 to 213) with known bacterial effectors with phosphothreonine lyase activity: OspF (*Shigella flexneri*), VirA (*Chromobacterium violaceum*), HopAI1 and HopAI1bis (*Pseudomonas syringae*) and SpvC (*S*. Typhimurium). The catalytic lysine residue of known phosphothreonine lyase proteins is indicated in the box. (F) HEK-293 cells transfected by pRK5myc-SpvD or pRK5myc-YopP were prepared at the indicated times after TNF-α stimulation and analysed by FACS using anti-myc and anti-IĸBα antibodies. Results are normalised to unstimulated cells, values are expressed as mean ± SEM of at least 3 independent experiments and compared to pRK5-myc (*p < 0.05; ** p < 0.01).
(TIF)

S5 Fig SpvD induces intra-nuclear accumulation of KPNA3 during infection. HeLa cells were cotransfected with FLAG-KPNA3 plasmids then infected for 14 h with *Salmonella* strains. Quantification of cells with nuclear lamina-associated KPNA3 after infection with *Salmonella* strains was assessed by microscopy. Values are expressed as mean ± SEM of at least 4 independent experiments (** p < 0.01; *** p < 0.005).
(TIF)

S6 Fig Xpo2 is required for importin recycling and p65 nuclear translocation. (A) Total HeLa cell levels of Xpo2 were analysed by immunoblotting in control cells or cells treated with oligo for Xpo2. Intracellular levels of GAPDH were used as a loading control. (B) HeLa cells depleted of Xpo2 (Xpo2 siRNA) or treated with scramble siRNA (scr. siRNA) and then transfected with FLAG-KPNA1 were fixed, labelled with anti-Xpo2 (blue), anti-FLAG (green). Cell nuclei were stained with DRAQ5 (red). Scale bar, 8 μm. (C) Localisation of KPNA1 in control cells (ctrl) or Xpo2 depleted (Xpo2 siRNA) was analysed by quantitative confocal immunofluorescence microscopy. Results are expressed as means ± SEM of 3 independent experiments and P-values were obtained using two-tailed unpaired Student's t-test (*** p < 0.005). (D) Representative immunofluorescence fields of p65 localisation using anti-p65 (red) in control cells or depleted of Xpo-2 (siRNA) after TNF-α stimulation (10 ng/ml) for 45 min. Cell nuclei were stained with DRAQ5 (green). Scale bar, 5 μm. (E) Quantification of p65 intensity in the nucleus was analysed by 3D confocal microscopy in cells with and without TNF-α treatment. Data were normalised to unstimulated cells in each condition (control and siRNA) and p65 nuclear translocation was expressed as the ratio of p65 intensity in the nucleus after stimulation compared to unstimulated cells. Results are expressed as means ± SEM of 3 independent experiments and P-values were obtained using two-tailed unpaired Student's t-test (* p < 0.05).
(TIF)

S7 Fig Cellular localisation of Xpo2 is not altered by SpvD. (A) HeLa cells were infected for 14 h with *Salmonella* strains. Cytoplasmic and total cell extracts were analysed by SDS-PAGE and immunoblotting with anti-Xpo2, anti-H3 and anti-GAPDH antibodies. (B) HeLa cells were transfected with vectors encoding myc-SpvD or myc-SpvC. Cytoplasmic and total cell extracts were analysed by SDS-PAGE and immunoblotting with anti-Xpo2, anti-H3 and anti-GAPDH antibodies.
(TIF)

S1 Table Levels of secreted TNF-α at 10 h post-uptake were quantified by ELISA in supernatants of *TLR4* ^-/-^ BMMs infected with indicated strains of *S*. Typhimurium. (DOCX)

S2 Table Bacterial strains and plasmids used in this study. (DOCX)

S3 Table Oligonucleotide primers used in this study. (DOCX)
