# Supplementary figures and images for: Inhibition of Nuclear Transport of NF-ĸB p65 by the Salmonella Type III Secretion System Effector SpvD
Source: PLoS Pathog. 2016 May 27;12(5):e1005653. doi: 10.1371/journal.ppat.1005653 (PMC4883751; doi:10.1371/journal.ppat.1005653)

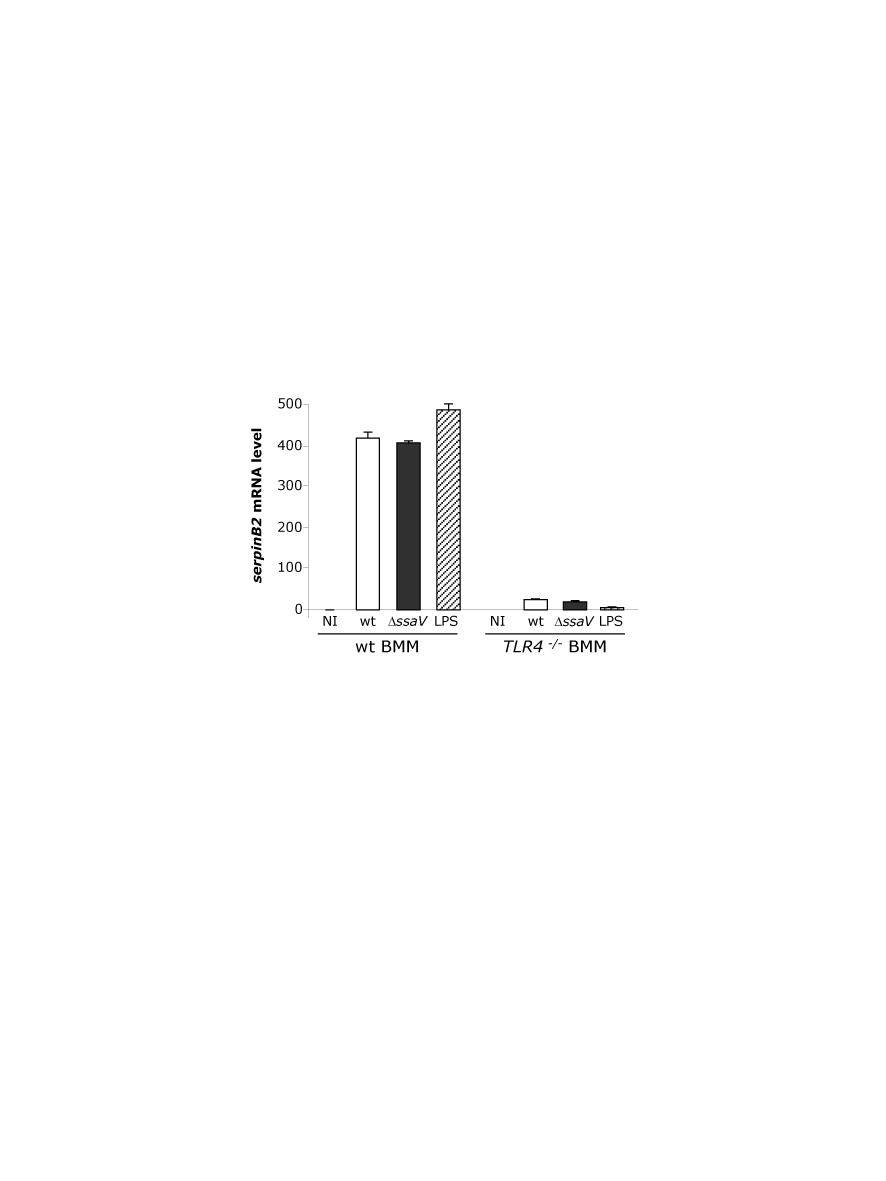

Supplement: S1 Fig — Wild-type and TLR4 -/- BMMs were non-infected (NI), exposed to LPS (100 ng/ml) or infected with wild-type or ΔssaV strains for 10 h and mRNA levels of serpinB2 were analysed by qRT-PCR after reverse transcription of RNA extracted from cells. The transcript levels were normalized to the levels of rsp9, which were constant under all conditions used, and then expressed relative to those of non-infected wild-type BMMs. Results are expressed as mean ± SEM of at least 3 independent experiments. (TIF) [file ppat.1005653.s001.tif]

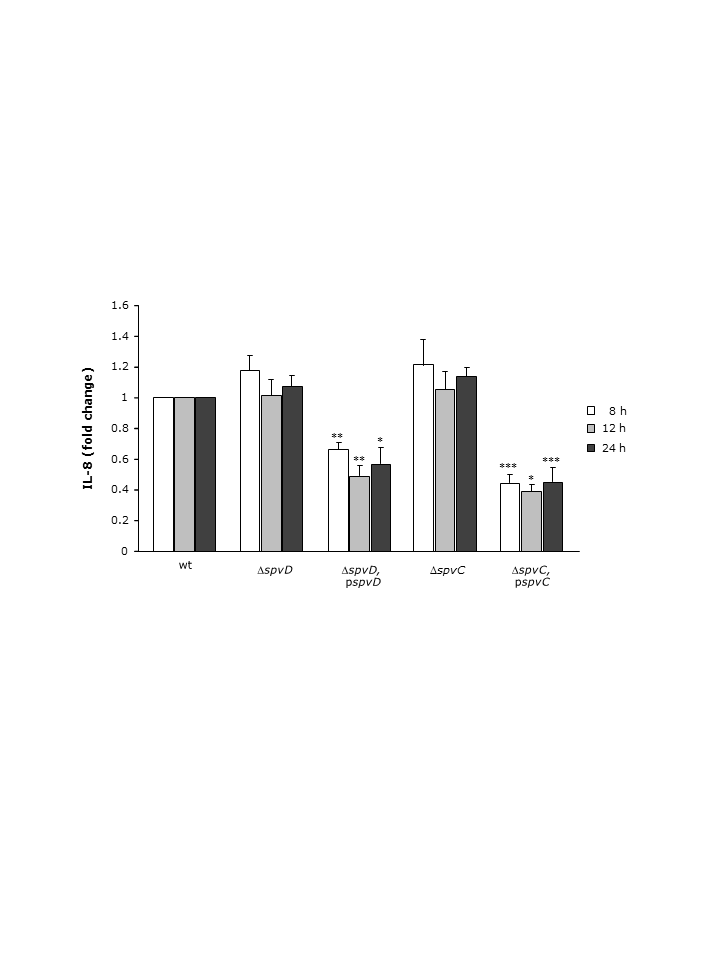

Supplement: S2 Fig — Levels of secreted IL-8 at 8 h, 12 h or 24 h post-uptake were quantified by ELISA in supernatants of HeLa cells, infected with indicated strains of S. Typhimurium. The cytokine levels were expressed relative to those of HeLa cells infected with wild-type bacteria. Results are expressed as means ± SEM of 3 independent experiments and P-values were obtained using two-tailed unpaired Student's t-test. (*p < 0.05; ** p < 0.01; *** p <0.005). (TIF) [file ppat.1005653.s002.TIF]

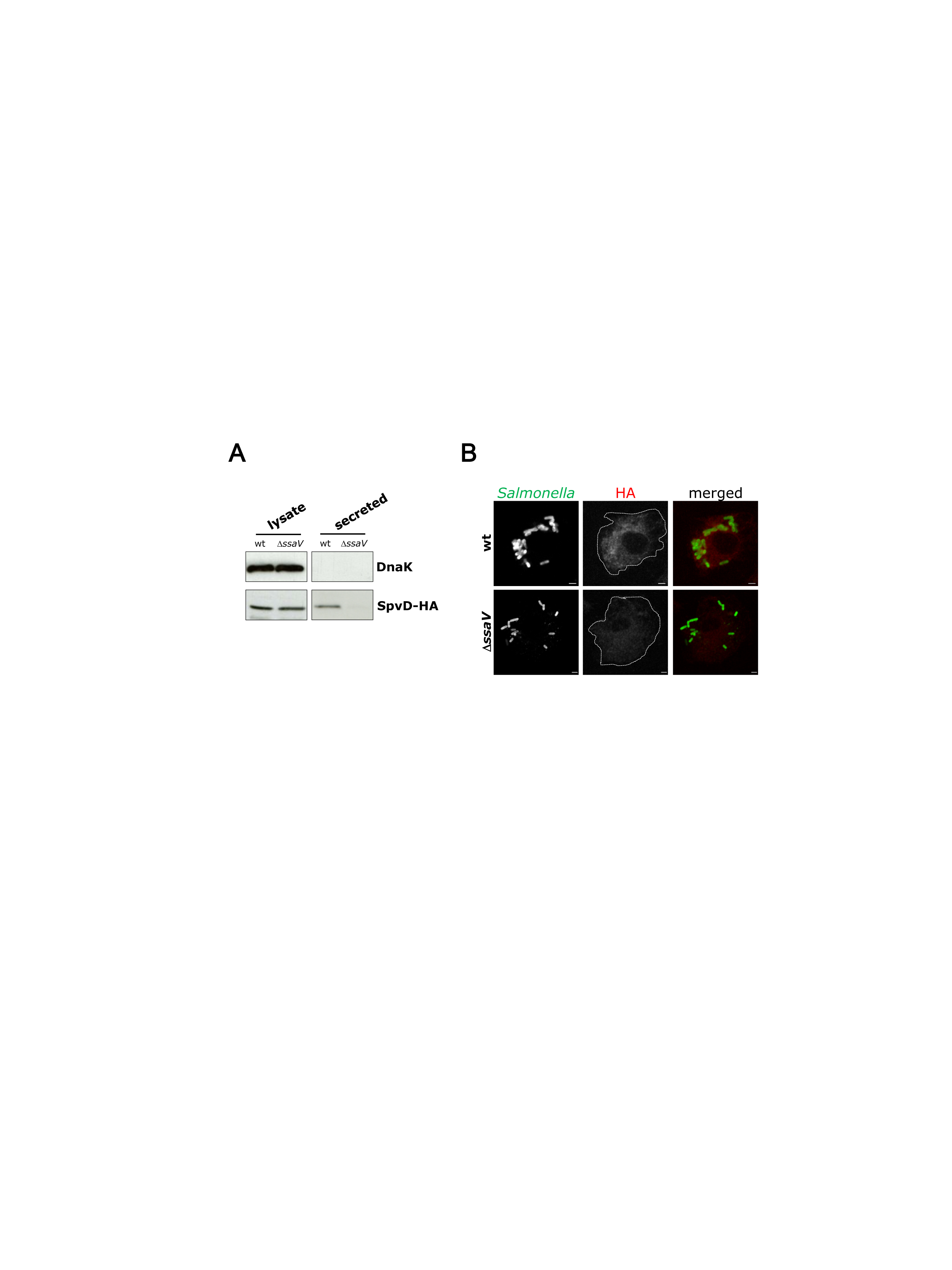

Supplement: S3 Fig — (A) Secretion of SpvD-2HA upon pH shift. Strains producing double-HA tagged SpvD from the virulence plasmid were used for pH shift analysis (as described in materials and methods). Secreted fractions and whole cell lysates were subjected to SDS-PAGE and immunoblotting. The intrabacterial protein DnaK was used as control. (B) RAW macrophages were infected for 24 h with Salmonella strains producing SpvD-2HA from the pSLT virulence plasmid; 10 μg/ml of MG132 was added to the culture media for the last 2 h of infection. Samples were fixed and labelled with antibodies against Salmonella and SpvD-2HA (green and red, respectively, on merged images). Scale bar, 2 μm. (TIF) [file ppat.1005653.s003.tif]

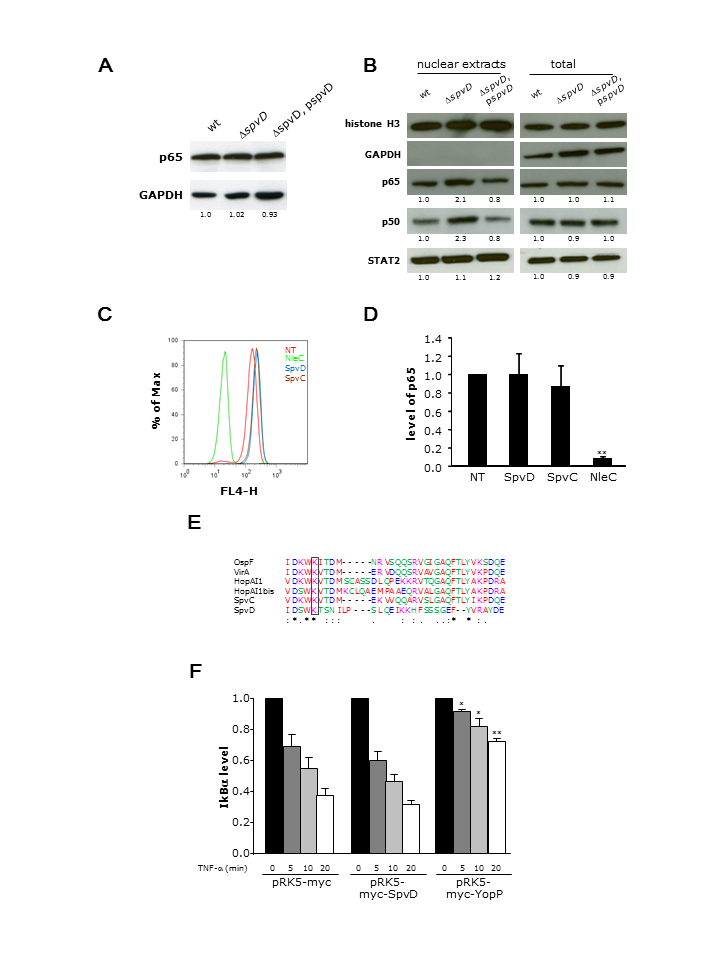

Supplement: S4 Fig — (A) TLR4 -/- BMMs were infected with different strains of Salmonella as indicated and p65 levels assessed by immunoblotting at 10 h post uptake. The same membrane was probed for GADPH as a loading control. Ratio of p65 normalised to wild-type-infected cells is indicated below immunoblots. (B) HeLa cells were infected for 14 h with Salmonella strains. Nuclear and total cell extracts were analysed by SDS-PAGE and immunoblotting with anti-histone H3, anti-GAPDH, anti-p65, anti-p50 and anti-STAT2 antibodies. Ratio of p65, p50 and STAT2 normalised to wild-type-infected cells are indicated below immunoblots. (C) Representative flow cytometry histogram of levels of p65 in HeLa cells non transfected (red) or transfected by pRK5myc-SpvD (blue), pRK5myc-SpvC (brown) or pRK5myc-NleC (green). (D) Quantification of p65 in cells expressing SpvD, SpvC or NleC. Data were normalised to non-transfected control cells (NT). Results are expressed as means ± SEM of 3 independent experiments and P-values were obtained using two-tailed unpaired Student's t-test. (** p < 0.01, compared to NT). (E) Alignment of SpvD C-terminal sequence (amino acids 181 to 213) with known bacterial effectors with phosphothreonine lyase activity: OspF (Shigella flexneri), VirA (Chromobacterium violaceum), HopAI1 and HopAI1bis (Pseudomonas syringae) and SpvC (S. Typhimurium). The catalytic lysine residue of known phosphothreonine lyase proteins is indicated in the box. (F) HEK-293 cells transfected by pRK5myc-SpvD or pRK5myc-YopP were prepared at the indicated times after TNF-α stimulation and analysed by FACS using anti-myc and anti-IĸBα antibodies. Results are normalised to unstimulated cells, values are expressed as mean ± SEM of at least 3 independent experiments and compared to pRK5-myc (*p < 0.05; ** p < 0.01). (TIF) [file ppat.1005653.s004.TIF]

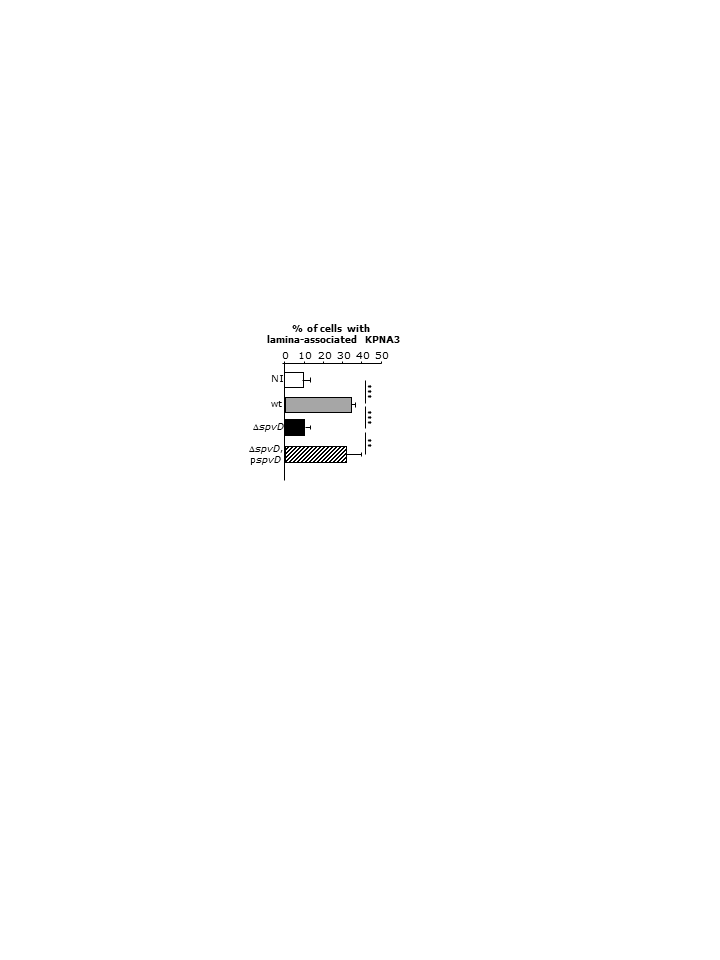

Supplement: S5 Fig — HeLa cells were cotransfected with FLAG-KPNA3 plasmids then infected for 14 h with Salmonella strains. Quantification of cells with nuclear lamina-associated KPNA3 after infection with Salmonella strains was assessed by microscopy. Values are expressed as mean ± SEM of at least 4 independent experiments (** p < 0.01; *** p < 0.005). (TIF) [file ppat.1005653.s005.tif]

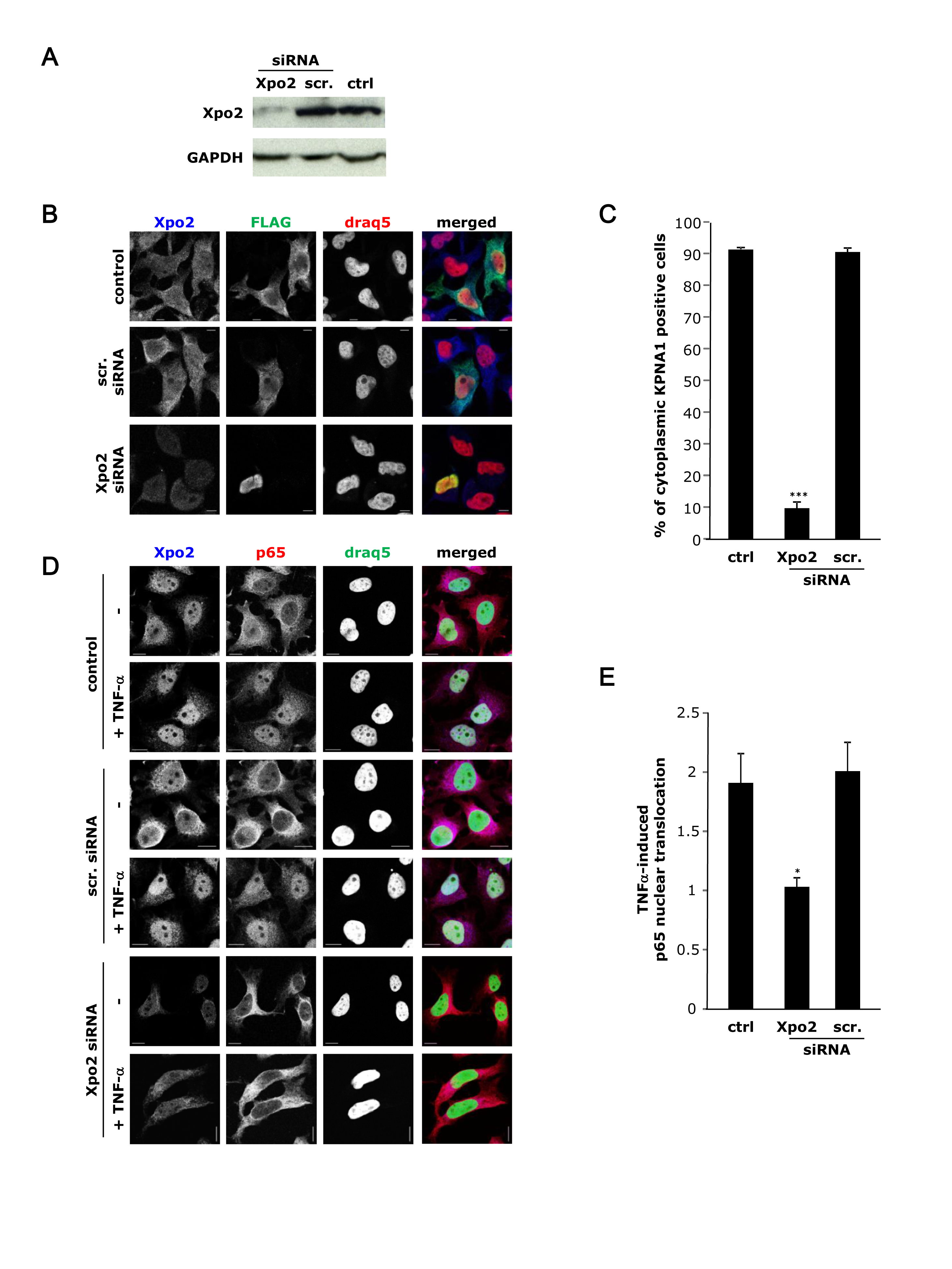

Supplement: S6 Fig — (A) Total HeLa cell levels of Xpo2 were analysed by immunoblotting in control cells or cells treated with oligo for Xpo2. Intracellular levels of GAPDH were used as a loading control. (B) HeLa cells depleted of Xpo2 (Xpo2 siRNA) or treated with scramble siRNA (scr. siRNA) and then transfected with FLAG-KPNA1 were fixed, labelled with anti-Xpo2 (blue), anti-FLAG (green). Cell nuclei were stained with DRAQ5 (red). Scale bar, 8 μm. (C) Localisation of KPNA1 in control cells (ctrl) or Xpo2 depleted (Xpo2 siRNA) was analysed by quantitative confocal immunofluorescence microscopy. Results are expressed as means ± SEM of 3 independent experiments and P-values were obtained using two-tailed unpaired Student's t-test (*** p < 0.005). (D) Representative immunofluorescence fields of p65 localisation using anti-p65 (red) in control cells or depleted of Xpo-2 (siRNA) after TNF-α stimulation (10 ng/ml) for 45 min. Cell nuclei were stained with DRAQ5 (green). Scale bar, 5 μm. (E) Quantification of p65 intensity in the nucleus was analysed by 3D confocal microscopy in cells with and without TNF-α treatment. Data were normalised to unstimulated cells in each condition (control and siRNA) and p65 nuclear translocation was expressed as the ratio of p65 intensity in the nucleus after stimulation compared to unstimulated cells. Results are expressed as means ± SEM of 3 independent experiments and P-values were obtained using two-tailed unpaired Student's t-test (* p < 0.05). (TIF) [file ppat.1005653.s006.tif]

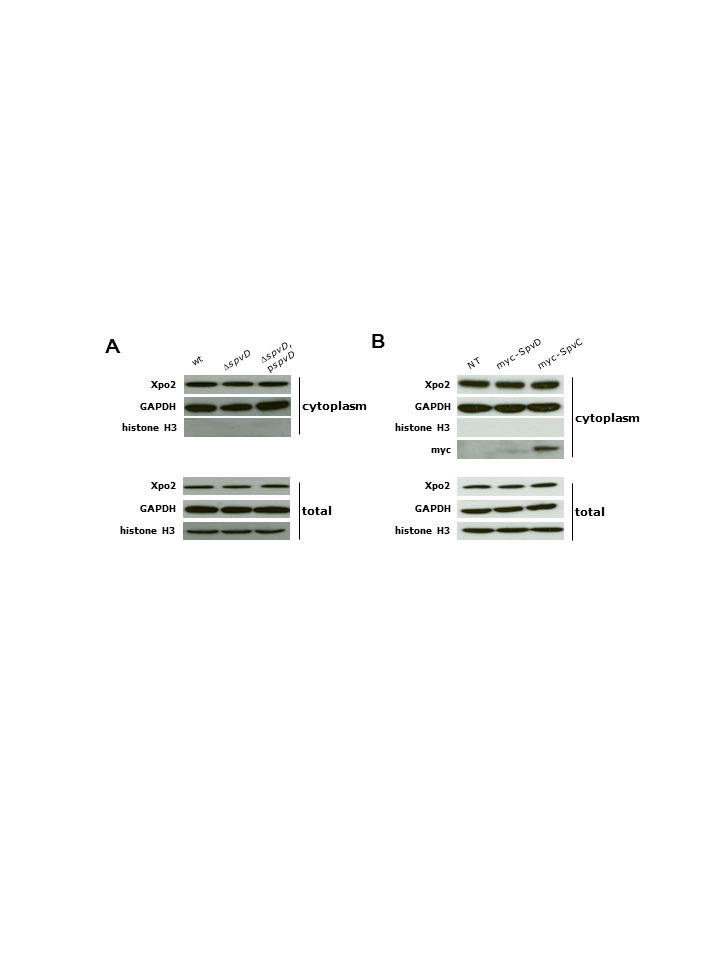

Supplement: S7 Fig — (A) HeLa cells were infected for 14 h with Salmonella strains. Cytoplasmic and total cell extracts were analysed by SDS-PAGE and immunoblotting with anti-Xpo2, anti-H3 and anti-GAPDH antibodies. (B) HeLa cells were transfected with vectors encoding myc-SpvD or myc-SpvC. Cytoplasmic and total cell extracts were analysed by SDS-PAGE and immunoblotting with anti-Xpo2, anti-H3 and anti-GAPDH antibodies. (TIF) [file ppat.1005653.s007.tif]
