# Supplementary material for: Inhibition of Nuclear Transport of NF-ĸB p65 by the Salmonella Type III Secretion System Effector SpvD
Source: PLoS Pathog. 2016 May 27;12(5):e1005653. doi: 10.1371/journal.ppat.1005653 (PMC4883751; doi:10.1371/journal.ppat.1005653)
Supplement: S1 Table — (DOCX) [file ppat.1005653.s008.docx]

**Table S1.** Levels of secreted TNF-α at 10 h post-uptake were quantified by ELISA in supernatants of *TLR4*^-/-^ BMMs infected with indicated strains of *S.* Typhimurium.

| ***Strains*** | ***TNF-α (fold change)*** | ***P-values*** |
| --- | --- | --- |
| wt | 1.00 ± 0.00 |  |
| Δ*ssaV* | 2.59 ± 0.63 | 0.012 |
| Δ*spvC* | 2.01 ± 0.40 | 0.018 |
| Δ*steC* | 1.99 ± 0.43 | 0.024 |
| Δ*spvD* | 1.61 ± 0.07 | 0.001 |
| Δ*sseJ* | 1.59 ± 0.15 | 0.003 |
| Δ*slrP* | 1.53 ± 0.06 | 0.001 |
| Δ*sspH1* | 1.46 ± 0.12 | 0.002 |
| Δ*sifB* | 1.49 ± 0.14 | 0.012 |
| Δ*avrA* | 1.37 ± 0.17 | 0.032 |
| Δ*steB* | 1.16 ± 0.29 | 0.091 |
| Δ*sopD2* | 1.15 ± 0.13 | 0.126 |
| Δ*srfJ* | 1.14 ± 0.11 | 0.089 |
| Δ*sseF* | 1.12 ± 0.09 | 0.113 |
| Δ*spvB* | 1.10 ± 0.11 | 0.124 |
| Δ*srfH* | 1.06 ± 0.13 | 0.190 |
| Δ*gogB* | 1.06 ± 0.18 | 0.171 |
| Δ*sopD* | 1.03 ± 0.25 | 0.104 |
| Δ*sseG* | 1.00 ± 0.09 | 0.200 |
| Δ*sifA* | 0.98 ± 0.28 | 0.156 |
| Δ*sptP* | 0.96 ± 0.13 | 0.370 |
| Δ*pipB2* | 0.95 ± 0.11 | 0.221 |
| Δ*steA* | 0.88 ± 0.19 | 0.089 |
| Δ*sseK1* | 0.86 ± 0.13 | 0.120 |
| Δ*pipB* | 0.84 ± 0.22 | 0.112 |
| Δ*sseK2* | 0.81 ± 0.25 | 0.172 |
| Δ*sseL* | 0.80 ± 0.10 | 0.063 |
| Δ*sspH2* | 0.72 ± 0.27 | 0.362 |

The cytokine levels were expressed relative to those of BMMs infected with wild-type (wt) bacteria. Results are expressed as mean ± SEM of at least 3 independent experiments. P-values were obtained using two-tailed unpaired Student’s t-test.
