# Supplementary material for: Inhibition of Nuclear Transport of NF-ĸB p65 by the Salmonella Type III Secretion System Effector SpvD
Source: PLoS Pathog. 2016 May 27;12(5):e1005653. doi: 10.1371/journal.ppat.1005653 (PMC4883751; doi:10.1371/journal.ppat.1005653)
Supplement: S2 Table — (DOCX) [file ppat.1005653.s009.docx]

**S2 Table.** Bacterial strains and plasmids used in this study

| **Name** | **Description** | **Reference** |
| --- | --- | --- |
| ***Strains*** |  |  |
| *S.* Typhimurium |  |  |
| wt | 12023 | NCTC |
| wt Km^R^ | STM0857 | [1] |
| Δ*ssaV* | strain HH109 (12023s *ssaV*::*aphT*) | [2] |
| Δ*sseB* | strain HH102 (12023s *sseB*::*aphT*) | [3] |
| Δ*sseB*, p*sseB* | Δ*sseB* harbouring pACYC*sseB* | [3] |
| Δ*spvD::*km | 12023 Δ*spvD::aphT* | [4] |
| Δ*spvD* | 12023 Δ*spvD* | this study |
| Δ*spvD*, p*spvD* | Δ*spvD* harbouring pACYC*spvD* | this study |
| Δ*spvC* | 12023 Δ*spvC* | [1] |
| Δ*spvC*, p*spvC* | Δ*spvC* harbouring pACYC*spvC*-2HA | [1] |
| Δ*spvRD* | 12023 Δ*spvR-spvA-spvB-spvC-spvD* | this study |
| Δ*spvRD*, p*spvD* | Δ*spvRD* harbouring pACYC*spvD* | this study |
| Δ*spvRD*, p*spvC* | Δ*spvRD* harbouring pACYC*spvC-2HA* | this study |
| *spvD*-2HA | 12023 *spvD*-2HA::Cm^R^ | this study |
| Δ*ssaV*, *spvD*-2HA | HH109 *spvD*-2HA::Cm^R^ | this study |
| Δ*spvD*, p*spvD*-2HA | Δ*spvD* harbouring pACYC*spvD*-2HA | this study |
| Δ*sseL*, p*sseL*-2HA | Δ*sseL* harbouring pWSK29*sseL*-2HA | [5] |
| Δ*steC* | 12023 Δ*steC*:km | [6] |
| Δ*sseJ* | 12023 Δ*sseJ*:cm | [7] |
| Δ*slrP* | 12023 Δ*slrP*:km | Andreas Bäumler |
| Δ*sspH1* | 12023 Δ*sspH1*:km | [4] |
| Δ*sifB* | 12023 Δ*sifB*:km | [4] |
| Δ*avrA* | 12023 Δ*avrA*:km | [4] |
| Δ*steB* | 12023 Δ*steB*:km | [4] |
| Δ*sopD2* | 12023 Δ*sopD2*:cm | [4] |
| Δ*srfJ* | 12023 Δ*srfJ*:km | [4] |
| Δ*sseF* | 12023 Δ*sseF*:km | [8] |
| Δ*spvB* | 12023 Δ*spvB*:km | [4] |
| Δ*srfH* | 12023 Δ*srfH*:km | [4] |
| Δ*gogB* | 12023 Δ*gogB*:km | [4] |
| Δ*sopD* | 12023 Δ*sopD*:km | [4] |
| Δ*sseG* | 12023 Δ*sseG*:km | [8] |
| Δ*sifA* | 12023 Δ*sifA*:km | [9] |
| Δ*sptP* | 12023 Δ*sptP*:km | [4] |
| Δ*pipB2* | 12023 Δ*pipB2*:km | [4] |
| Δ*steA* | 12023 Δ*steA*:km | [4] |
| Δ*sseK1* | 12023 Δ*sseK1*:km | [4] |
| Δ*pipB* | 12023 Δ*pipB*:km | [4] |
| Δ*sseK2* | 12023 Δ*sseK2*:km | [4] |
| Δ*ssseL* | 12023 Δ*sseL*:km | [5] |
| Δ*sspH2* | 12023 Δ*sspH2*:km | [4] |
| *E. coli* |  |  |
| EPEC E2348/69 | wt | NCTC |
|  |  |  |
| ***Plasmids*** |  |  |
| pKD4 | PCR template plasmid with Km resistance cassette, Km^R^ | [10] |
| pKD46 | plasmid encoding arabinose-inducible λ-Red recombinase system, Amp^R^ | [10] |
| pCP20 | plasmid encoding arabinose-inducible FLP recombinase, Amp^R^ | [11] |
| pSU315 | Rep_R6K_ 2HA FRT, Km^R^ | [12] |
| pACYC184 | Rep_p15A_ low copy number vector | [13] |
| pGD2::myc-*yopP* | myc fused to YopP in pEF6 | [14] |
| p*spvD* | pACYC184 with *spvD* cloned into *EcoRV* and *SalI* sites, Cm^R^ | this study |
| p*spvD*-2HA | pACYC184 with C-terminal 2HA-tagged *spvD* cloned into *EcoRV* and *SalI* sites, Cm^R^ | this study |
| pRK5-myc | eukaryotic expression vector | lab stock |
| pRK5-myc-SpvD | pRK5 with N-terminal myc-tagged SpvD cloned into *BamHI* and *EcoRI* sites, Amp^R^ | this study |
| pRK5-myc-SpvD_K185A_ | pRK5 with N-terminal myc-tagged SpvD_K185A_ cloned into *BamHI* and *EcoRI* sites, Amp^R^ | this study |
| pRK5-myc-SpvC | pRK5 with N-terminal myc-tagged SpvC cloned into *BamHI* and *EcoRI* sites, Amp^R^ | this study |
| pRK5-myc-NleC | pRK5 with N-terminal myc-tagged NleC cloned into *BamHI* and *EcoRI* sites, Amp^R^ | this study |
| pRK5-myc-YopP | pRK5 with N-terminal myc-tagged YopP cloned into *NotI* and *EcoRI* sites, Amp^R^ | this study |
| M_3_psinrevκB-*luc* | *luc* gene under control of NF-ĸB consensus promoter | gift from F. Randow |
| pRLTK | constitutively active Renilla luciferase | gift from F. Randow |
| pAP-1-luc | *luc* gene under control of AP-1 consensus promoter | gift from J. Kagan |
| pcDNA3.1*Xpo5*-FLAG | pcDNA3.1 with C-terminal FLAG-tagged Xpo5 | [13] |
| pcDNA3.1*Xpo2*-FLAG | pcDNA3.1 with C-terminal FLAG-tagged Xpo2 | this study |
| pcCAGGS*KPNA1*-FLAG | pcCAGGS with C-terminal FLAG- tagged KPNA1 | [15] |
| pcCAGGS*KPNA3*-FLAG | pcCAGGS with C-terminal FLAG- tagged KPNA3 | [15] |

**References**

1. Mazurkiewicz P, Thomas J, Thompson JA, Liu M, Arbibe L, et al. (2008) SpvC is a Salmonella effector with phosphothreonine lyase activity on host mitogen-activated protein kinases. Mol Microbiol 67: 1371-1383.

2. Deiwick J, Nikolaus T, Shea JE, Gleeson C, Holden DW, et al. (1998) Mutations in Salmonella pathogenicity island 2 (SPI2) genes affecting transcription of SPI1 genes and resistance to antimicrobial agents. J Bacteriol 180: 4775-4780.

3. Hensel M, Shea JE, Waterman SR, Mundy R, Nikolaus T, et al. (1998) Genes encoding putative effector proteins of the type III secretion system of Salmonella pathogenicity island 2 are required for bacterial virulence and proliferation in macrophages. Mol Microbiol 30: 163-174.

4. Figueira R, Watson KG, Holden DW, Helaine S (2013) Identification of salmonella pathogenicity island-2 type III secretion system effectors involved in intramacrophage replication of S. enterica serovar typhimurium: implications for rational vaccine design. MBio 4: e00065.

5. Rytkonen A, Poh J, Garmendia J, Boyle C, Thompson A, et al. (2007) SseL, a Salmonella deubiquitinase required for macrophage killing and virulence. Proc Natl Acad Sci U S A 104: 3502-3507.

6. Poh J, Odendall C, Spanos A, Boyle C, Liu M, et al. (2008) SteC is a Salmonella kinase required for SPI-2-dependent F-actin remodelling. Cell Microbiol 10: 20-30.

7. Ruiz-Albert J, Yu XJ, Beuzon CR, Blakey AN, Galyov EE, et al. (2002) Complementary activities of SseJ and SifA regulate dynamics of the Salmonella typhimurium vacuolar membrane. Mol Microbiol 44: 645-661.

8. Kuhle V, Hensel M (2002) SseF and SseG are translocated effectors of the type III secretion system of Salmonella pathogenicity island 2 that modulate aggregation of endosomal compartments. Cell Microbiol 4: 813-824.

9. Beuzon CR, Salcedo SP, Holden DW (2002) Growth and killing of a Salmonella enterica serovar Typhimurium sifA mutant strain in the cytosol of different host cell lines. Microbiology 148: 2705-2715.

10. Datsenko KA, Wanner BL (2000) One-step inactivation of chromosomal genes in Escherichia coli K-12 using PCR products. Proc Natl Acad Sci U S A 97: 6640-6645.

11. Cherepanov PP, Wackernagel W (1995) Gene disruption in Escherichia coli: TcR and KmR cassettes with the option of Flp-catalyzed excision of the antibiotic-resistance determinant. Gene 158: 9-14.

12. Uzzau S, Figueroa-Bossi N, Rubino S, Bossi L (2001) Epitope tagging of chromosomal genes in Salmonella. Proc Natl Acad Sci U S A 98: 15264-15269.

13. Chang WL, Tarn WY (2009) A role for transportin in deposition of TTP to cytoplasmic RNA granules and mRNA decay. Nucleic Acids Res 37: 6600-6612.

14. Denecker G, Declercq W, Geuijen CA, Boland A, Benabdillah R, et al. (2001) Yersinia enterocolitica YopP-induced apoptosis of macrophages involves the apoptotic signaling cascade upstream of bid. J Biol Chem 276: 19706-19714.

15. Shaw ML, Cardenas WB, Zamarin D, Palese P, Basler CF (2005) Nuclear localization of the Nipah virus W protein allows for inhibition of both virus- and toll-like receptor 3-triggered signaling pathways. J Virol 79: 6078-6088.
