# Supplementary material for: Inhibition of Nuclear Transport of NF-ĸB p65 by the Salmonella Type III Secretion System Effector SpvD
Source: PLoS Pathog. 2016 May 27;12(5):e1005653. doi: 10.1371/journal.ppat.1005653 (PMC4883751; doi:10.1371/journal.ppat.1005653)
Supplement: S3 Table — (DOCX) [file ppat.1005653.s010.docx]

**S3 Table.** Oligonucleotide primers used in this study

| **Primer** | **Sequence (5’-3’)** | **Description** |
| --- | --- | --- |
| Del spvR  Del spvD | CCTGTTTTTGCACATCAAAACATTTTTTCAGGATTATTCTGAAAAAAAAAAGGAGATATTGTGTAGGCTGGAGCTGCTTC  GGATCTTGCTAAGGCTCTCTATTAACTTACCATTCATAAAATGAATATTTAAAAAAGTTACATATGAATATCCTCCTTAG | primers to amplify Km^R^ cassette from pKD4 for making deletion of the entire operon (genes *spvR* to *spvD*) |
| pacyDF  pacyDR | GGGGGATATCTAGGTAGTGTAACTATGAGA  CGCCCGTCGACTTTTAAAAAAGTTATCAATC | for cloning *spvD* from the virulence plasmid into pACYC184 using *EcoRV* and *SalI* sites |
| pacyDF  pacyDR-2HA | GGGGGATATCTAGGTAGTGTAACTATGAGA  CGCCCGTCGACTTACTAGAGGCTAGCATAATCAGGAACATCATACGGATACGCATAGTCCGGCACATCATACGGATAATCGTGTTTTTCATCATAAGCCCT | for cloning *spvD-2HA* from the virulence plasmid into pACYC184 with an C-terminal 2HA-tag using *EcoRV* and *SalI* sites |
| SpvD 2HA 1  SpvD 2HA 2 | AGGGGAATTTTATGTCAGGGCTTATGATGAAAAACACGATTATCCGTATGATGTGCCGGACTATGCGTATCCGTATGATGTTCCTGATTATGCTAGCCTCTAGTAA  CCTGCCCTAAAGCCCTTCGGGCTTTTCGCCTTATATCCCCCATATGAATATCCTCCTTAGT | for 2HA tagging of SpvD on the virulence plasmid by insertion of Km^R^ cassette amplified from pSU315 upstream of *spvD* |
| pRK5-SpvC1  pKR5-SpvC2 | CGGGATCCATGCCCATAAATAGGCCTAATC  CGGAATTCTTACTCTGTCATCAAACGATA | for cloning *spvC* from the virulence plasmid into pRK5-myc using *BamHI* and *EcoRI* sites |
| pRK5-SpvD1  pKR5-SpvD2 | CGGGATCCATGAGAGTTTCTGGTAGTGCG  CGGAATTCTCAATCGTGTTTTTCATCATA | for cloning *spvD* from the virulence plasmid into pRK5-myc using *BamHI* and *EcoRI* sites |
| pRK5-NleC1  pRK5-NleC2 | CGGGATCCATGAAAATTCCCTCATTACAG  CGGAATTCTCATCGCTGATTGTGTTTGTC | for cloning *nleC* from EPEC into pRK5-myc using *BamHI* and *EcoRI* sites |
| pRK5-Yop1  pRK5-Yop2 | ATAAGAATGCGGCCGCATGATCGGACCAATATCACA  CGGAATTCTTATACTTTGAGAAGTGTTT | for cloning *yopP* from pGD2::myc-*yopP*  into pRK5-myc using *NotI* and *EcoRI* sites |
| SpvD_K185A_ 1  SpvD_K185A_ 2 | AAGTAACTAAGAACCCGATGTAGGTTGTATGAAGGATCG  TCCTTCATACAACCTACATCGGGTTCTTAGTTACTTAAC | for site directed mutagenesis |
| CAS NheI  CAS XhoI | CCTAGCTAGCTATGGAACTCAGCGATGCAAATCTG  CCGCCTCGAGAAGCAGTGTCACACTGGCTGCC | for cloning *Xpo2* into pcDNA3.1 using *NheI* and *XhoI* sites |
| rsp9 1  rsp9 2 | CTGGACGAGGGCAAGATGAAGC  TGACGTTGGCGGATGAGCACA | for qRT-PCR |
| il-1β 1  il-1β 2 | CCAGCCAAGCTTCCTTGTGC  CAGCCCAGGTCAAAGGTTTGG | for qRT-PCR |
| tnf-α 1  tnf-α 2 | GGCAGGTCTACTTTGGAGTCA  ACATTCGAGGC TCCAGTGAAT | for qRT-PCR |
| serpinB2 1  serpinB2 1 | AACACTGAACAGCAGATGGC  AGAGAGGAGAAGGCTGAATG | for qRT-PCR |
